# Modulating Activity through Defect Engineering of Tin Oxides for Electrochemical CO_2_ Reduction

**DOI:** 10.1002/advs.201900678

**Published:** 2019-07-04

**Authors:** Rahman Daiyan, Emma Catherine Lovell, Nicholas M. Bedford, Wibawa Hendra Saputera, Kuang‐Hsu Wu, Sean Lim, Jonathan Horlyck, Yun Hau Ng, Xunyu Lu, Rose Amal

**Affiliations:** ^1^ Particles and Catalysis Research Laboratory School of Chemical Engineering The University of New South Wales Sydney NSW 2052 Australia; ^2^ Department of Chemical Engineering Institut Teknologi Bandung Bandung 40132 Indonesia; ^3^ Electron Microscope Unit The University of New South Wales Sydney NSW 2052 Australia; ^4^ School of Energy and Environment City University of Hong Kong Hong Kong China

**Keywords:** CO_2_ reduction, defect engineering, flame spray pyrolysis, formate, oxygen hole centers, oxygen vacancy, SnO_2_

## Abstract

The large‐scale application of electrochemical reduction of CO_2_, as a viable strategy to mitigate the effects of anthropogenic climate change, is hindered by the lack of active and cost‐effective electrocatalysts that can be generated in bulk. To this end, SnO_2_ nanoparticles that are prepared using the industrially adopted flame spray pyrolysis (FSP) technique as active catalysts are reported for the conversion of CO_2_ to formate (HCOO^−^), exhibiting a FE_HCOO_^−^ of 85% with a current density of −23.7 mA cm^−2^ at an applied potential of −1.1 V versus reversible hydrogen electrode. Through tuning of the flame synthesis conditions, the amount of oxygen hole center (OHC; Sn≡O●) is synthetically manipulated, which plays a vital role in CO_2_ activation and thereby governing the high activity displayed by the FSP‐SnO_2_ catalysts for formate production. The controlled generation of defects through a simple, scalable fabrication technique presents an ideal approach for rationally designing active CO_2_ reduction reactions catalysts.

## 1 Introduction

Solar powered electrochemical CO_2_ reduction reactions (CO_2_RR), to high value liquid organic products such as formate, is a potential strategy to address the rising CO_2_ concentration in the atmosphere and at the same time provide a feasible alternative to store the intermittent renewable energy within the current infrastructure.^1^, ^2^ There is a considerable economic demand for generating formate as it is widely utilized as a chemical feedstock in the pharmaceutical and textile industry and recently invoked as an energy source in generating electricity in fuel cells.^3^, ^4^, ^5^ Despite the development of numerous catalyst materials for CO_2_RR to formate, the large‐scale adoption of this technology remains limited by the absence of high‐performing inexpensive electrocatalysts that can be produced in bulk and that requires lower energy input.^6^, ^7^ To date inexpensive foil‐based catalysts for CO_2_RR to formate (such as Sn, Bi, and Pb)^8^, ^9^, ^10^, ^11^, ^12^, ^13^, ^14^, ^15^ exhibit low conversion and require high applied overpotentials. Thus, research has focused on the quest to make scalable active catalysts by developing novel nanostructures that can promote mass transport, present higher electrical conductivity as well as contain more active sites. Nevertheless, the fabrication of these high performing electrocatalysts (specifically Sn‐based catalysts which are nontoxic and abundant) for formate generation still require the adoption of intricate fabrication techniques such as plasma treatments, nanocasting, rapid quenching, and encapsulation in carbon‐based supports and some of the highly reported activity can only be achieved with the aid of performance boosting techniques such as high electrolyte pH, IR compensation, high mass loading, and gas diffusion electrode (GDE) systems.^16^, ^17^, ^18^, ^19^, ^20^ As such, it is becoming imperative to develop designer SnO_2_ catalysts using scalable methods that would allow control over tuning the active sites for CO_2_RR. In addition to developing catalysts with suitable active sites (for higher selectivity), it is also of utmost importance to improve the binding of CO_2_ reactants on electrocatalysts (for lowering overpotential as well as enhancing overall conversion) as this step is widely reported as one of the limiting factors for CO_2_RR.^21^, ^22^ This can be achieved through the introduction of defects within the catalysts and such vacancy defects are reported to improve CO_2_ activation.^23^, ^24^ While oxygen vacancy defects were ascribed as beneficial for CO_2_RR to formate in prior reports,^23^, ^25^ to the best of our knowledge there exists no systematic investigation tuning these vacancies and associating the nature of such defects as well as correlation with CO_2_RR. In this regard, the use of a facile technique to systematically vary defect sites to improve reactant binding for selective CO_2_RR to formate is desired.

Here, we report SnO_2_ nanoparticles prepared using the commercialized flame spray pyrolysis (FSP) process as a viable catalyst for the electrocatalytic reduction of CO_2_ to formate. The high temperature facilitated by the FSP process allows precise control over the formation of nonstoichiometric atomic arrangements and vacancies that are commonly ascribed as beneficial for heterogeneous catalysis.^26^, ^27^ In this work, we tuned the precursor feed rate during FSP, varying the flame temperature and fuel equivalence ratio in order to enable significant alteration in the particle size along with the surface defect sites. This systematic and controlled variation in surface defects, including oxygen vacancies and oxygen hole centers, correlates strongly with the CO_2_RR activity. Our extensive ex situ characterization (electron paramagnetic resonance (EPR), X‐ray absorption spectroscopy (XAS), and high‐energy X‐ray diffraction (HE‐XRD) coupled to pair distribution function (PDF) analysis) as well as in situ characterization (Raman) reveal that apart from the traditionally ascribed active sites, surface defects play a crucial role in activating CO_2_ on Sn‐based catalysts, providing further insights into designing cost‐effective electrocatalysts for CO_2_RR.

## 2 Results and Discussion

In order to systematically control the morphology and surface properties of SnO_2_, FSP was utilized. By varying the precursor feed rate to the flame, the flame conditions can be controlled and thus allow for the tuning of material properties such as particle size and morphology, crystallinity, and defects.^26^, ^28^ In this work, the FSP‐SnO_2_ catalysts were synthesized at a feed‐rate of 3, 5, and 7 mL min^−1^ and are denoted as FSP‐SnO_2_‐3, FSP‐SnO_2_‐5, and FSP‐SnO_2_‐7, respectively (detailed in the Experimental Section).

**Figure 1:**
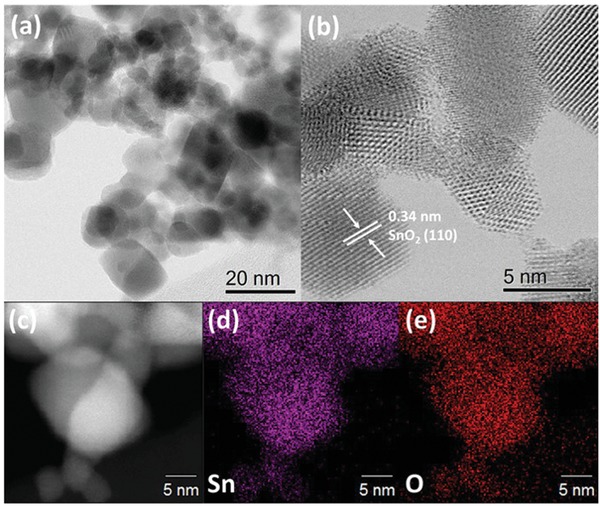
HR‐TEM imaging and EDX mapping of FSP‐SnO_2_‐5.

**Figure** **1** displays the high‐resolution transmission electron microscopy images (HR‐TEM) of the FSP‐synthesized SnO_2_ (at 5 mL min^−1^ precursor feed rate). It is evident that the FSP results in the formation of highly crystalline (Figure 1b), nonporous, spheroid particles with a broad distribution of particle sizes. Lattice fringes with a spacing of ≈ 0.34 nm, exemplify the presence of SnO_2_ (110) facets within the material.^29^ Further, the energy dispersive spectroscopy (EDX) mapping of the catalyst also demonstrated a uniform presence of Sn and O. The variation in surface morphology, as a result of the increasing precursor feed rate during SnO_2_ preparation, is clearly evident in TEM images (Figure S1, Supporting Information). It can be observed that a higher feed‐rate during FSP results in increasing particle sizes (from ≈9 to ≈14 nm as the feed rate increases from 3 to 7 mL min^−1^) of the FSP‐SnO_2_ which is consistent with the X‐ray diffraction (XRD) and N_2_ adsorption‐desorption results, vide infra.

**Figure 2:**
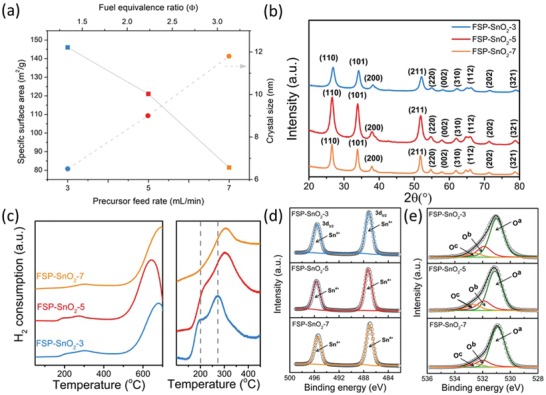
Physiochemical properties of FSP‐SnO_2_ catalysts. a) Impact of precursor feed rate (and fuel equivalence ratio, *Φ*) on specific surface area and crystallite size, b) XRD patterns, c) TPR profiles and high‐resolution XPS spectra showing d) Sn 3d and e) O 1s of FSP‐SnO_2_‐3, FSP‐SnO_2_‐5 and FSP‐SnO_2_‐7. O^a^, O^b^, and O^c^ refer to peaks corresponding to Sn^4+^‐O, adsorbed oxygen and O atoms adjacent to oxygen vacancies, respectively.

As shown in **Figure** **2**a, the increase in precursor feed rate resulted in a clear decrease in Brunauer–Emmett–Teller (BET) specific surface area (from 146 m^2^ g^−1^ for FSP‐SnO_2_‐3 to 81.4 m^2^ g^−1^ for FSP‐SnO_2_‐7) with a corresponding increase in the BET equivalent diameter, *d*_BET_ (from 5.9 nm for FSP‐SnO_2_‐3 to 10.6 nm for FSP‐SnO_2_‐7, Table S1, Supporting Information). This increase in surface area is consistent with the literature and arises from the changing flame conditions.^26^ Typically, increasing the precursor feed rate results in an increase in fuel being fed to the flame, an increase in the metal concentration within the flame as well as longer residence times at higher temperature.^30^, ^31^ All of this contributes to increased particle size growth (and thus decreased specific surface areas). It should be noted that the N_2_ adsorption‐desorption isotherms (Figure S2, Supporting Information) indicate an IUPAC Type III isotherm consistent with a nonporous material.^32^ The presence of hysteresis may indicate capillary condensation within pores, however as FSP produces nonporous materials (as confirmed with TEM), this is likely arising from inter‐particle voids. The bulk crystallinity of the FSP‐SnO_2_ samples obtained was then analyzed with the aid of XRD (Figure 2b). From Figure 2b, it is clear that tetragonal rutile SnO_2_ phase is present for all the FSP‐synthesized SnO_2_. This is indicated by the peaks at 2θ of 26.6°, 33.8°, and 51.8°, corresponding to (110), (101), and (310) facets of SnO_2_ respectively (JCPDS 41‐1445).^33^ The Scherrer equation was employed to estimate the crystallite sizes of the catalysts (Figure 2a) and the results were found to be consistent with the BET and TEM results (Table S1, Supporting Information).

The reduction behavior of SnO_2_ was investigated using hydrogen temperature programmed reduction (H_2_‐TPR). As indicated in Figure 2c, the H_2_‐TPR profiles exhibit two distinct major peaks, one at low temperature region (200–300 °C) that corresponds to reduction of the SnO_2_ surface and possibly subsurface, and at high temperature region (640–700 °C) that can be ascribed to the reduction of the bulk SnO_2_.^34^ Provided that the catalyst surface plays a major role in electrocatalysis, we carried out a closer inspection of the low temperature reduction peaks. An additional secondary peak (at ≈200 °C) for FSP‐SnO_2_‐3 is evident, as well as a shoulder for FSP‐SnO_2_‐5. The decreasing intensity of this additional peak with increasing feed rate during FSP clearly suggests that increasing feed‐rate leads to an increase in oxygen vacancy (facilitated by a decrease in complete oxidation) and this phenomenon can be explained using the fuel equivalence ratio. The fuel equivalence ratio, *Φ*, displayed in Figure 2a, is a measure of the portion of the required oxygen for complete combustion of the supplied solutions to the actual oxygen flow rate (supplied from the dispersion gas).^31^ This clearly indicates the anticipated increase in oxygen deficiency as feed rate increases, which implies less available oxygen for complete oxidation. To understand the surface chemical composition, X‐ray photoelectron spectroscopy (XPS) was utilized. As indicated by the survey spectra (Figure S3, Supporting Information), only peaks corresponding to Sn, O, and C can be identified. Figure 2d displays the Sn 3d spectra, indicating that the surface species in all the FSP‐SnO_2_ catalysts are Sn^4+^, as only two major peaks can be observed at binding energy of 487.6 and 496.8 eV corresponding to Sn 3d_5/2_ and Sn 3d_3/2_, respectively.^35^, ^36^ The high‐resolution O1s spectra (Figure 2e) can be deconvoluted into three peaks at binding energy ≈531 eV (referred as O^a^), ≈532 eV (O^b^), and ≈533 eV (O^c^) corresponding to the presence of Sn^4+^‐O, adsorbed oxygen and O atoms adjacent to oxygen vacancies, respectively.^35^ It was also discovered that the surface oxygen species varied amongst the catalysts (Figure S4, Supporting Information), with higher feed rate resulting in decreased adsorbed O and that oxygen vacancy defects was maximized for FSP‐SnO_2_‐5. Collectively, these characterization results indicate the controlled systematic tuning of the physiochemical properties of SnO_2_, empowering us to undertake a thorough investigation into the role of SnO_2_ as well as defects in defining CO_2_RR performance. A clear, and anticipated trend, with decreasing surface area with increasing precursor feed rate was evident along with an increase in oxygen vacancies.

**Figure 3:**
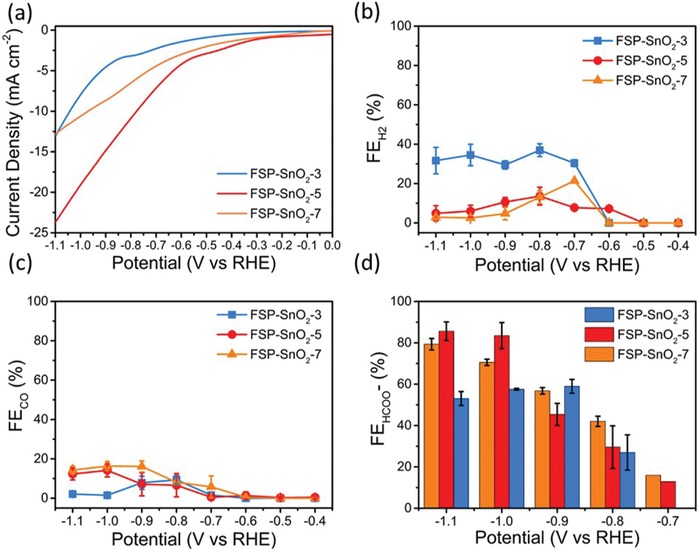
Electrocatalytic performance of FSP‐SnO_2_ catalysts in CO_2_ saturated 0.1 m KHCO_3_. a) Linear sweep voltammetry (scan rate: 5 mV s^−1^) and dependence of b) ${\mathrm{FE}}_{H_{2}}$, c) FE_CO_, and d) FE_HCOO_^−^ with applied potential for FSP‐SnO_2_‐3, FSP‐SnO_2_‐5, and FSP‐SnO_2_‐7 in CO_2_ saturated 0.1 m KHCO_3_ solution.

The activity of FSP‐SnO_2_ toward CO_2_RR was evaluated using 2 h long potentiostatic studies carried out in CO_2_ saturated 0.1 m KHCO_3_. The polarization curves, presented in **Figure** **3**a, clearly demonstrate that the current density (*j*) attained with FSP‐SnO_2_‐5 was higher compared to FSP‐SnO_2_‐3 and FSP‐SnO_2_‐7 electrodes. At the applied potential of −1.1 V, the attained *j* with FSP‐SnO_2_‐5 was −23.7 mA cm^−2^ compared to −13.3 and −12.9 mA cm^−2^ observed with FSP‐SnO_2_‐3 and FSP‐SnO_2_‐7. Moreover, two gas phase products (H_2_ and CO) were detected using gas chromatography and the dependence of the Faradaic efficiency for H_2_ $\left({\mathrm{FE}}_{H_{2}}\right)$ and CO (FE_CO_) on the applied potential is illustrated in Figure 3b,c, respectively. It is evident that increasing precursor feed rate resulted in an overall decrease in ${\mathrm{FE}}_{H_{2}}$ (at −1.1 V, ${\mathrm{FE}}_{H_{2}}$ with FSP‐SnO_2_‐3, FSP‐SnO_2_‐5, and FSP‐SnO_2_‐7 was measured to be 31%, 5%, and 3%, respectively), which is beneficial as more charge can be consumed toward CO_2_RR rather than the competing HER.^37^ In addition, we observe that the production of CO is also suppressed with the FSP catalysts reported herein. The maximum FE_CO_ attained with FSP‐SnO_2_‐3, FSP‐SnO_2_‐5, and FSP‐SnO_2_‐7 was 8%, 13%, and 17% at −0.7, −1.0, and −1.0 V, respectively. Nuclear magnetic resonance (NMR) revealed that the only liquid product generated during CO_2_RR was the target formate (HCOO^−^) with all the FSP catalysts exhibiting a strong potential dependence for FE_HCOO_^−^. Of these catalysts, FSP‐SnO_2_‐5 was the most active toward CO_2_RR to formate, requiring an onset overpotential of 375 mV whilst both FSP‐SnO_2_‐3 and FSP‐SnO_2_‐7 required 575 and 475 mV of additional overpotential before formate was detected. At all potentials, FSP‐SnO_2_‐5 exhibited better selectivity toward HCOO^−^ production, attaining a maximum FE_HCOO_^−^ of 85% at −1.1 V whereas the maximum FE_HCOO_^−^ attained with FSP‐SnO_2_‐3 and FSP‐SnO_2_‐7 were 60% and 79% at −0.9 and −1.1 V, respectively. We also carried out mechanistic Tafel analysis (Figure S5, Supporting Information) and found that the Tafel slope for FSP‐SnO_2_‐5 is much smaller, indicating faster reaction kinetics on the catalyst for CO_2_RR. The overall enhanced catalytic activity as well as notable stability (Figure S6, Supporting Information) of FSP‐SnO_2_‐5 is amongst the highest for CO_2_RR to formate (Table S2, Supporting Information), thereby endorsing its suitability for potential large‐scale applications.

Combining the structure‐performance results for the FSP catalysts, it is clear that the particle size/surface area variation of the catalysts (as a result of the varying feed rates) is not governing the overall production rate for the FSP catalysts for formate production as the catalyst with the higher surface area (i.e., FSP‐SnO_2_‐3) does not demonstrate a higher partial current density for formate production (*j*_HCOO_^−^, Figure S7, Supporting Information). To further validate this, volumetric CO_2_ adsorption measurements were carried out (Figure S8, Supporting Information), which clearly demonstrated that physical CO_2_ adsorption on the catalysts do not dictate the CO_2_RR selectivity trends. Interestingly, both XRD and electrochemical impedance spectroscopy (EIS, Figure S9 in the Supporting Information) measurements suggested similar conductivity of the FSP‐SnO_2_ catalysts and thereby cannot be used to explain the CO_2_RR trends outlined above. The above results alongside the electrochemical surface area measurements (ECSA, Figure S10 in the Supporting Information) reveal the physical changes of the FSP catalysts do not have a direct correlation with their varied selectivity.

**Figure 4:**
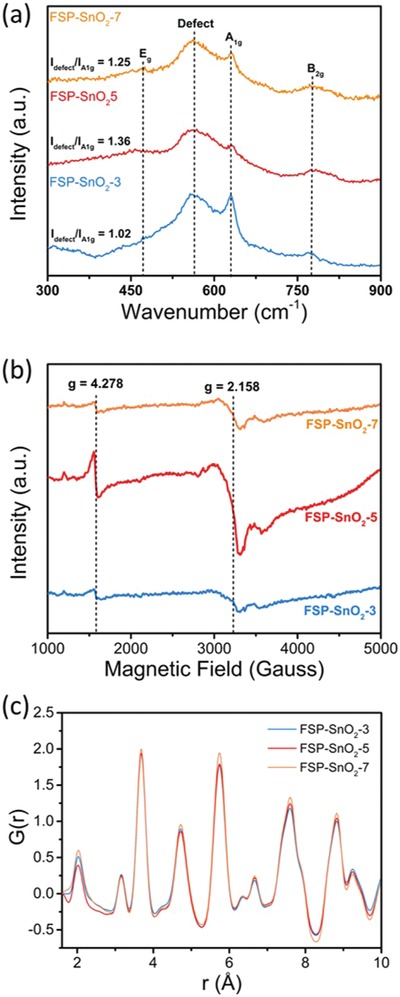
Defect identification of FSP‐SnO_2_ catalysts. a) Raman spectra at 514 nm, b) EPR spectra at 120 K, c) Fourier transforms for atomic pair distribution function for FSP SnO_2_ prepared at a feed rate of 3, 5, and 7 mL min^−1^.

After ruling out the physical properties, the role of chemical state and electronic properties of the catalysts in dictating CO_2_RR was systematically studied. From the TPR profiles (Figure 2c), it was clear that the increasing feed rate during FSP led to a reduction in surface oxygen species (as a result of incomplete combustion). Our XPS results further indicated that defects arising from oxygen vacancies were more prominent for FSP‐SnO_2_‐5, suggesting the potential role of such sites in facilitating CO_2_RR to formate. These defect sites are reported to govern the adsorption of CO_2_ reactants near the active sites, enabling higher conversion at lower applied overpotentials.^23^, ^38^ To confirm the formation of surface defects, Raman spectroscopy was carried out with the FSP‐SnO_2_ catalysts where the defects present within the different FSP‐catalysts were probed. The Raman spectra for FSP‐SnO_2_ catalysts (**Figure** **4**a) demonstrated peaks at wavenumber 474, 627, and 768 cm^−1^ that correspond to the E_g_, A_1g_, and B_2g_ vibration modes, respectively. These are in good agreement with rutile bulk SnO_2_ reported in literature.^39^, ^40^ Additionally, a peak at ≈575 cm^−1^ assigned to surface defects present in SnO_2_ was detected.^39^ It can be observed from Figure 4a, the intensity ratio between defects and the resonant A_1g_ phonon (*I*_defect_/*I* ${}_{A_{\mathrm{1g}}}$) was maximized for FSP‐SnO_2_‐5 (*I*_defect_/*I* ${}_{A_{\mathrm{1g}}}$ = 1.36) followed by FSP‐SnO_2_‐7 (*I*_defect_/*I* ${}_{A_{\mathrm{1g}}}$ = 1.25) and the lowest was for FSP‐SnO_2_‐3 (*I*_defect_/*I* ${}_{A_{\mathrm{1g}}}$ = 1.02). These results are consistent with the trends observed with CO_2_RR where FSP‐SnO_2_‐5 demonstrated the highest activity. UV–vis carried out with FSP‐SnO_2_‐5 further indicated that these defects are not changing the electronic states of the catalyst (Figure S11, Supporting Information).^39^, ^41^ Electron paramagnetic resonance was also utilized to semi‐quantitatively determine the defects in the bulk of FSP‐SnO_2_ catalysts. It can be seen from Figure 4b that two major EPR peaks at *g* values of 2.158 and 4.278 are observed which can be attributed to the formation of oxygen hole centers (OHC; Sn≡O●, where “≡” denotes the three Sn—O bond and “●” is the unpaired electron) and Sn^3+^ associated with oxygen vacancy species, respectively.^42^ These OHC defects can be formed by cleaving Sn–O–Sn linkages or by the rupture of a Sn‐OH group,^43^ while Sn^3+^ associated with oxygen vacancy can be generated through incomplete oxidation during FSP synthesis.^44^ It has been reported that OHC sites, alongside the radicals, assists in the binding of CO_2_ reactants,^25^, ^38^, ^45^ as well as active sites to stabilize the formate anion radical intermediate.^23^ These OHC defects contain additional electrons which can be attained by the adjacent metal sites or be trapped in the vacancy sites, rather than become delocalized over the whole surface.^46^, ^47^ The surface of the electrocatalyst would therefore be electron‐rich and these excess electrons facilitate CO_2_ adsorption as well as lowering the activation energy for CO_2_RR.^23^, ^24^ As a consequence, we can ascribe the catalytic activity of the FSP‐SnO_2_ catalysts with the amount of OHC defects. The electrocatalytic results show a direct dependence on the amounts of defects (semi‐quantitatively measured using double‐integrated intensity of EPR spectra, Figure S12, Supporting Information). For instance, FSP‐SnO_2_‐5 with the highest OHC defects demonstrated the highest FE_HCOO_^−^ followed by FSP‐SnO_2_‐7 with the second highest concentration of OHC defects and lastly FSP‐SnO_2_‐3 which contained the lowest amount of OHC defects. To further confirm the role of these defects, we carried out a control experiment where the active FSP‐SnO_2_‐5 was thermally annealed in air (referred as HT‐FSP‐SnO_2_‐5) to reduce the amount of OHC. This control sample was then evaluated for CO_2_RR (Figure S13, Supporting Information) and we can clearly demonstrate that the removal of defects (as observed from EPR spectra and the corresponding double integrated intensity, Figure S14, Supporting Information) leads to decreased formate production, in line with our hypothesis.

Additionally, we investigated the electronic configuration of the FSP catalysts with the aid of X‐ray absorption spectroscopy. The X‐ray absorption near‐edge spectra (XANES) showcase very similar edge features for all FSP samples that are characteristic of SnO_2_ (Figure S15, Supporting Information), which strongly suggests that the majority of the Sn share similar electronic configuration. The lack of sensitivity to defect structures in the XANES (as well as EXAFS) is likely due to a combination of core‐hole broadening that occurs in heavier elements and relative concentration of surface defects in material.^48^ Likewise, the Fourier transformed EXAFS spectra are also similar with the FSP‐SnO_2_ catalysts, suggesting that a similar atomic environment is shared among the Sn species (Figures S16–S18, Supporting Information). The fitted EXAFS results (Table S3, Supporting Information) for the FSP‐SnO_2_ catalysts clearly indicate that the coordination number is ≈5.1 for the oxygen first‐shell, with a fitted bond length of 2.06 Å. These results collectively suggest that the defects within FSP‐SnO_2_ are on the surface of the catalysts and thereby the low concentration is not easily observable from Sn K‐edge EXAFS measurements.

Additional synthesis‐dependent structural information was obtained using atomic pair distribution function analysis (Figure 4c). Atomic PDFs are derived from the FT of high‐energy XRD patterns (Figures S19 and S20, Supporting Information) and provide structural information on the basis of all atomic pairs in the material weighted toward their relative concentration and electron density at distances approaching the length scale of nanoscale materials.^49^, ^50^, ^51^ Atomic PDFs provide more versatility in understanding longer range structures than EXAFS (which are limited to <5 Å) as these function arise from scattering data and are not directly influenced by modifications to electronic structure. From Figure 4c, all samples showcase characteristic atomic pairs associated with the known crystal structure of SnO_2_, with minor structural variations found throughout the PDF. The relative magnitude of the Sn–O first coordination sphere suggests disordered surface structure caused by defect formation that may not have been directly observed via EXAFS.^52^ Moreover, the relative dampening of PDF oscillations toward higher atomic pair distances for FSP‐SnO_2_‐7 indicates a higher degree of longer‐range order under these synthetic conditions (Figure S21, Supporting Information), likely due to a decrease in defects and an overall increase in particle size. Crystallographic modeling techniques were implemented to quantify structural changes that occur between the samples synthesized under different FSP conditions (see the Experimental Section for details). The resulting *a* and *c* lattice parameters in Table S4 (Supporting Information) showcase a general trend to unit cell expansion with increasing feed rate, from 4.7213 Å for FSP‐SnO_2_‐3 to 4.7234 Å for FSP‐SnO_2_‐7 in the *a* lattice parameter, corresponding well with the formation of oxygen vacancies which are reported to cause lattice expansion.^53^, ^54^ Interestingly, the *c* lattice parameter shows less of a change with FSP feed rate, with values of 3.1774, 3.1780, and 3.1772 Å for FSP‐SnO_2_‐3, FSP‐SnO_2_‐5, and FSP‐SnO_2_‐7 respectively. In addition to lattice constant changes, anisotropic temperature factors can be used to better understand how defects influence overall structure, as a defective moiety represents an increase in atomic position uncertainty from a crystallographic viewpoint. Shown in Table S5 (Supporting Information), the anisotropic temperature factors for O atoms are ≈5× bigger than for Sn atoms, showcasing the impact of formation of O defects and terminal O atoms at the catalyst surface.^55^ It can be observed from Table S5 (Supporting Information) that the FSP‐SnO_2_‐5 exhibited the highest O anisotropic temperature factor of 0.04221, followed by FSP‐SnO_2_‐3 and FSP‐SnO_2_‐7 (0.04166 and 0.03852 respectively). While being sensitive to defects, anisotropic temperature factors are inversely related to nanoparticle size as well, as an increase in surface atoms become crystallographically dissimilar from the bulk lattice. Given that the surface area decreases between FSP‐SnO_2_‐3 and FSP‐SnO_2_‐5, the increase in anisotropic temperature factor between the catalysts is evidence of greater formation of defects within FSP‐SnO_2_‐5. In contrast, FSP‐SnO_2_‐7 exhibited a large decrease in anisotropic temperature factor as a result of the lowest surface area presented by the catalyst (albeit the catalyst demonstrated a slight decrease in defects compared to FSP‐SnO_2_‐5).

It is well established that the application of bias during CO_2_RR with SnO_2_ catalysts leads to partial reduction of oxide layers to generate SnO*_x_* sites.^56^, ^57^ The removal of O from defected SnO_2_ lattice during in situ reduction of the catalyst results in increasing the oxygen vacancies. To understand this changing defect structure under applied potentials, in situ Raman spectroscopy (detailed in Figure S22, Supporting Information) was carried out with FSP‐SnO_2_‐5. In the absence of an applied potential, the Raman spectra (Figure S23, Supporting Information) for the catalyst demonstrated peaks typical for SnO_2_ vibration modes as well as that of surface defects (consistent with Figure 4a, vide supra).These peaks were retained after the application of negative bias during CO_2_RR, validating the presence of SnO_2_ (in conformity with post‐reacted XRD pattern and XPS spectra of FSP‐SnO_2_‐5 electrode, Figures S24 and S25, Supporting Information) and defects during CO_2_RR.

The collective structure–activity relationship for the FSP‐SnO_2_ catalysts demonstrate a clear dependence of selectivity toward HCOO^−^ on the defects (characterized by XPS, Raman, semi‐qualitatively by EPR, XAS, and PDF), highlighting for the first time the modulation of CO_2_RR activity through defect engineering. The collective characterization results confirm the formation of highest amounts of defects in FSP‐SnO_2_‐5 (**Table** **1** and Figure S26, Supporting Information), translating into very high overall CO_2_RR activity of the catalyst. It is clear that increasing feed rate resulted in a decrease in surface area. This increasing feed rate also resulted in an increase in oxygen vacancies (as a result of an increase in the oxygen deficiency of the flame). Thus, the maxima of surface defects of the FSP‐SnO_2_‐5 sample can be attributed to the tradeoff between the surface area and oxygen vacancies. Ultimately, these factors play a synergistic role to maximize the surface defects on FSP‐SnO_2_‐5. For instance, FSP‐SnO_2_‐3 contained a smaller amount of defects (indicated by Raman and EPR) but demonstrated a high anisotropic temperature factor because of its higher surface area. However, in the case of FSP‐SnO_2_‐5, the surface area decreases but we observe a maximum for the anisotropic temperature factor which is suggesting a significantly higher amount of defects (further corroborated by the highest *I*_D_/*I*_G_ ratio and EPR signal). A further increase in feed‐rate (i.e., FSP‐SnO_2_‐7) is found to lead to a slight decrease in defects but a much larger decrease in anisotropic temperature factor as a result of lower surface area exhibited by FSP‐SnO_2_‐7. On the basis of the above findings, it is suggested that the CO_2_ reactant molecules are initially adsorbed on the defects present on the surface of the optimized FSP‐SnO_2_, allowing the subsequent conversion of CO_2_ to formate on the SnO_2_ active sites. The presence of defects is known to increase the charge density along the valence band maximum, improving CO_2_ activation (as the localized electrons are transferred to the antibonding orbitals of CO_2_ reactants),^24^, ^58^ thereby allowing higher conversion of CO_2_ to formate as well as lowering onset overpotentials. Most importantly, these findings illustrate the ability to potentially tune CO_2_RR properties through synthetic manipulation of FSP conditions to optimizing for surface area and defect density.

**Table 1:** Defect–activity relationship for FSP‐catalysts prepared at a feed rate of 3, 5, and 7 mL min^−1^. The Faradic efficiency toward formate (FE_HCOO_^−^) was obtained at an applied potential of −1.1 V. Defect densities were calculated through Raman (*I*_defect_/*I* ${}_{A_{\mathrm{1g}}}$), EPR (OHC double integrated intensity), and PDF (O anisotropic temperature factor)

Catalyst	FE_HCOO_^−^ [%]	*I*_defect_/*I* ${}_{A_{\mathrm{1g}}}$	OHC double integrated intensity [a.u.]	O Anisotropic temperature factor
FSP‐SnO_2_‐3	51	1.02	0.012	0.0417
FSP‐SnO_2_‐5	85	1.36	0.032	0.0422
FSP‐SnO_2_‐7	79	1.25	0.014	0.0385

## 3 Conclusion

In summary, we have developed and demonstrated an effective approach for scalable fabrication and implementation of defective SnO_2_ catalysts for the large‐scale CO_2_ reduction to formate. Through extensive characterizations, we have revealed the decisive role of OHC in activating CO_2_ reactants, allowing higher activity toward formate production. Through control of the fabrication technique, we were able to modulate the amount of the defects, allowing us to achieve a high a FE_HCOO_^−^ of 85% with a current density of −23.7 mA cm^−2^ at an applied potential of −1.1 V with the FSP‐SnO_2_ catalysts.

## 4 Experimental Section

*Catalyst Preparation*: Three SnO_2_ samples were prepared via flame spray pyrolysis in a setup described previously.^31^ A solution of tin 2‐ethylhexanoate (Sigma Aldrich, 92.5‐100%) in xylene was prepared such that the total Sn concentration was 0.5 m. This solution was fed to the FSP flame at three different flow rates (3, 5, and 7 L min^−1^) with a syringe pump, to give three separate SnO_2_ samples. The precursor solution was dispersed by a constant 5 L min^−1^ flow of oxygen (Coregas, >99.9%). The flame was ignited and maintained with a supporting flame mixture which consisted of 3.2 L min^−1^ oxygen and 1.5 L min^−1^ methane (Coregas, >99.95%). The flame was directed with the aid of a 5 L min^−1^ flow of oxygen and a vacuum pump toward a glass fiber filter, where the SnO_2_ nanoparticles were deposited and collected.

*Electrochemical Reduction of CO_2_ Measurements*: Catalyst inks were prepared by dispersing 5 mg of FSP‐SnO_2_ catalysts in 0.5 mL deionized water and ethanol solution (1:1, v/v) followed by the addition of 25 µL of Nafion solution (Sigma‐Aldrich). The sonicated homogenous inks were then drop‐casted on carbon fiber paper (loading: 1 mg cm^−2^) to yield the working electrodes. The working electrodes were then placed with a saturated calomel reference electrode (SCE) in the cathodic compartment of a customized gas‐tight H‐cell whereas the anodic compartment consisted of a Pt wire as the counter electrode. The two compartments of the H‐cell were separated by a glass frit to ensure that the reduction products from the cathode did not oxidize in the anode. The electrolyte utilized in this study was 0.1 m KHCO_3_ and prior to every experiment, the cathodic part of the H‐cell was purged with CO_2_ for duration of 30 min and the pH of the saturated 0.1 m KHCO_3_ solution was 6.8. All potentials measured in this study were converted to the reversible hydrogen electrode (RHE) reference for the purpose of comparison using the following equation: *E*_RHE_ (V) = *E*_SCE_ (V) + 0.245 + 0.059 × pH. The electrochemical measurements were carried out using a CHI 760E (CH Instrument, Texas) electrochemical workstation. Potentiostatic studies were carried out at various potentials for a duration of 2 h and the experiments repeated and the results presented are the averaged values. For each potential, the results for the first hour of the first testing is discarded as the working electrode required conditioning.

*Product Detection*: The gas phase products were quantified after 3600 and 7200 s during the 2 h electrolysis session using a gas chromatograph (Shimidzu, Model 2010 Plus) equipped with both thermal conductivity detector (TCD) and flame ionization detector (FID) detectors. To detect the liquid products, 0.5 mL of aliquot was collected at the end of each bulk electrolysis at fixed potentials and was mixed with 0.1 mL of D_2_O and 7.143 ppm of internal standard dimethyl sulfoxide (DMSO, Sigma 99.99%) and was analyzed using a 600 MHz ^1^H 1 D liquid NMR spectrometer (Bruker Advance) at 25 °C. The 1D ^1^H spectrum was measured with water suppression with a pre‐saturation method (representative spectra displayed in Figure S27, Supporting Information). The amounts of products were calculated by comparing the integral areas of the observed formate product with that of the DMSO. The peak positions were calibrated using formic acid (HCOOH, 98%, Sigma Aldrich) dissolved in 0.1 m KHCO_3_ solution containing the internal standard solution.

*Physical Characterization*: Surface morphology of the catalysts were studied using TEM with a Phillips CM 200 microscopy that was operated at 200 kV. Surface chemical composition was evaluated using X‐ray photoelectron spectroscopy with a Thermo ESCALAB250i X‐ray photoelectron spectrometer. UV–vis absorption spectra of the samples were acquired using a Shimadzu UV‐3600 UV–vis–NIR spectrophotometer with BaSO4 as the reference. The structural characterization was studied using powder X‐Ray Diffraction pattern using PANalytical X'Pert instrument using Cu K_∝_ radiation (λ = 1.54 Å) and scan range from 10° to 90°. BET isotherms were measured on a Micrometrics Tristar 3030 using nitrogen adsorption at 77 K. Raman spectroscopy was carried out using Renishaw inVia Raman Microscope (514 nm laser). In situ Raman spectra were carried out with FSP‐SnO_2_‐5 catalyst drop‐casted on glassy carbon electrode and was placed in CO_2_ saturated 0.1 m KHCO_3_ solution in the custom cell where SCE reference and Pt counter electrode were utilized. Raman spectra was first obtained when no potential was applied to establish the background. Subsequently, Raman measurements were carried out when the applied potential was −1.5 V (for 300 s) versus RHE to study the change in surface chemical states for the catalyst during CO_2_RR. The presence of defects was evaluated using electron paramagnetic resonance spectroscopy on a Bruker EMX‐plus X‐Band EPR spectrometer. The EPR measurements were conducted at 9.41 GHz (X‐band) at 120 K with the microwave power set at 2 mW and the modulation amplitude at 5G. Hydrogen temperature programmed reduction measurements were conducted using a Micromeritics Autochem II 2920 instrument to study reducibility of the SnO_2_ surface and bulk. ≈50 mg of sample was placed into a U‐shaped quartz sample tube, on a plug of quartz wool, and heated to 150 °C under 20 mL min^−1^ Ar (Coregas Argon, >99.99%) at a heating rate of 10 °C min^−1^ and held for 30 min. Following pre‐treatment, the sample was cooled to 50 °C. The TPR analysis was conducted under a 10% H_2_‐Ar (20 mL min^−1^) flow with the temperature ramped from 50 to 700 °C at 10 °C min^−1^. High‐energy X‐ray diffraction experiments were performed at the 11‐IB‐B beamline of the advanced photon source (APS). Powders were loaded into Kapton capillaries and examined using 86.7 keV X‐rays. A sample to detector distance of 220 mm was used to obtain a *q*_max_ of ≈32 Å^−1^, which as calibrated with a reference CeO_2_ powder. HE‐XRD patterns were background corrected (Figure S18, Supporting Information), transformed into reduced structure factors (Figure S19, Supporting Information), and Fourier transformed into atomic PDFs using the program RAD.^59^ Crystallographic modeling was performed with PDFgui using the known bulk crystal structure for SnO_2_.^60^, ^61^ X‐ray absorption spectroscopy experiments were performed at the 10‐ID‐B beamline of the APS. SnO_2_ powders were uniformly spread across Kapton tape and examined using a fluorescence geometry. Spectra were obtained from 200 eV below the Sn K‐edge (29.2 keV) up to 1000 eV past the edge. Subsequent data processing and EXAFS modeling was performed using the Demeter software package.^62^ For EXAFS modeling (Figure S17, Supporting Information), Sn–O and Sn–Sn backscattering contributions were obtained using the same reference lattice for PDF modeling and a S_0_^2^ value of 0.879 as obtained from modeling a metallic Sn reference foil.

## Conflict of Interest

The authors declare no conflict of interest.

## Supporting information

Supplementary
