# Supplementary material for: Modulating Activity through Defect Engineering of Tin Oxides for Electrochemical CO2 Reduction
Source: Adv Sci (Weinh). 2019 Jul 4;6(18):1900678. doi: 10.1002/advs.201900678 (PMC6755522; doi:10.1002/advs.201900678)
Supplement: Supplementary file 1 — Supplementary [file ADVS-6-1900678-s001.pdf]

Copyright WILEY-VCH Verlag GmbH & Co. KGaA, 69469 Weinheim, Germany, 2019.

ADVANCED SCIENCE
Open Access

Supporting Information

for *Adv. Sci.,* DOI: 10.1002/advs.201900678

Modulating Activity through Defect Engineering of Tin Oxides for Electrochemical CO_2 Reduction

Rahman Daiyan, Emma Catherine Lovell, Nicholas M. Bedford, Wibawa Hendra Saputera, Kuang-Hsu Wu, Sean Lim, Jonathan Horlyck, Yun Hau Ng, Xunyu Lu, and Rose Amal**

Supporting Information

Modulating Activity through Defect Engineering of Tin Oxides for Electrochemical CO_2 Reduction

Rahman Daiyan[†,1] *Emma Catherine Lovell*[†,1] *Nicholas M. Bedford,*[1] *Wibawa Hendra Saputera,*[1,2] *Kuang Hsu Wu,*[1] *Sean Lim,*[3] *Jonathan Horlyck,*[1] *Yun Hau Ng,*[4] *Xunyu Lu*,*[1] *Rose Amal**[1]

[1] Particles and Catalysis Research Laboratory, School of Chemical Engineering, The University of New South Wales, Sydney, NSW 2052, Australia

[2] Department of Chemical Engineering, Institut Teknologi Bandung, Bandung 40132, Indonesia

[3] Electron Microscope Unit, The University of New South Wales, Sydney, NSW 2052, Australia

[4] School of Energy and Environment, City University of Hong Kong, Hong Kong, China

Corresponding Author
*Rose Amal (r.amal@unsw.edu.au)
*Xunyu Lu (xunyu.lu@unsw.edu.au)

[†] **R.D. & E.C.L contributed equally.**

Supplementary Figures

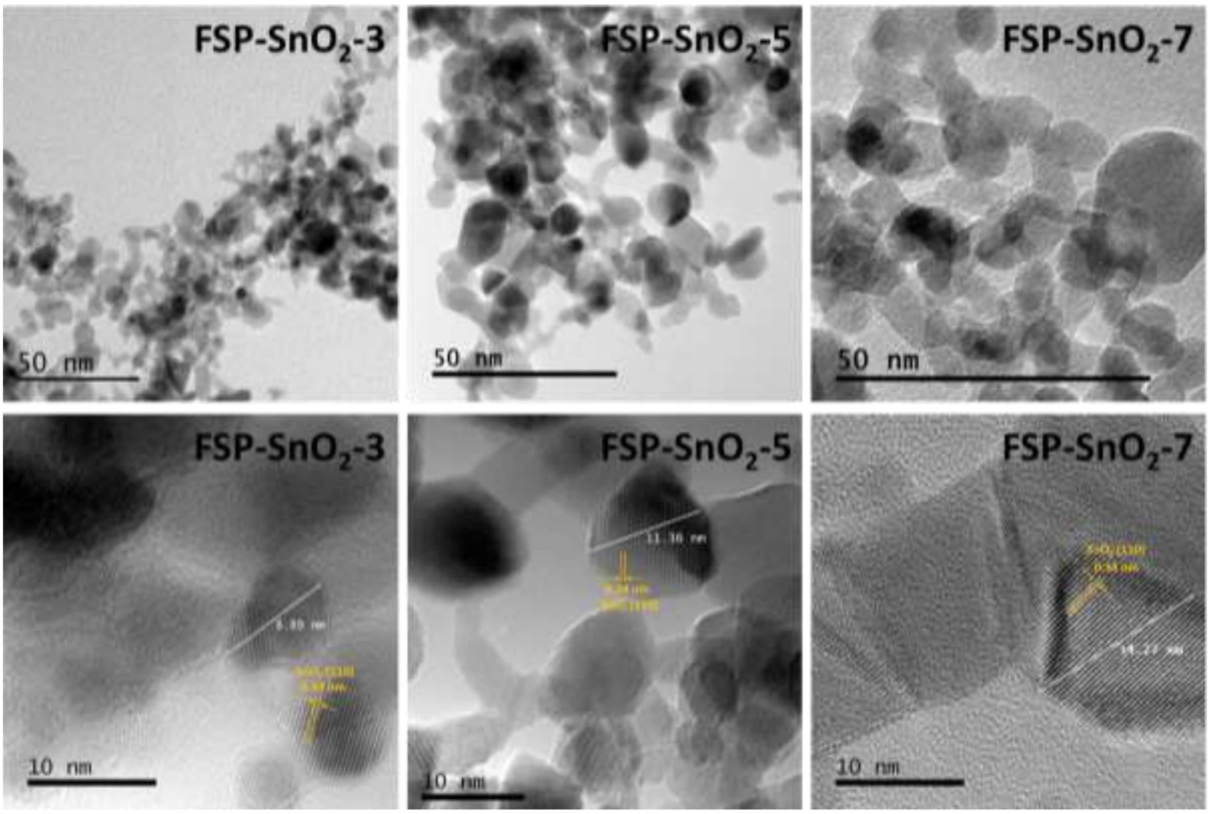

Figure S1. TEM images of FSP-SnO_2 catalysts prepared at different feed rates. From the TEM images it is clear that increasing feed rate during FSP results in particle sizes increasing from 9 nm (FSP-SnO_2-3) to 11 nm (FSP-SnO_2-5) to 14 nm (FSP-SnO_2-7). Additionally, with all the catalysts, a lattice fringe of ~ 0.34 nm that corresponds to (110) facets of SnO_2 is observed.

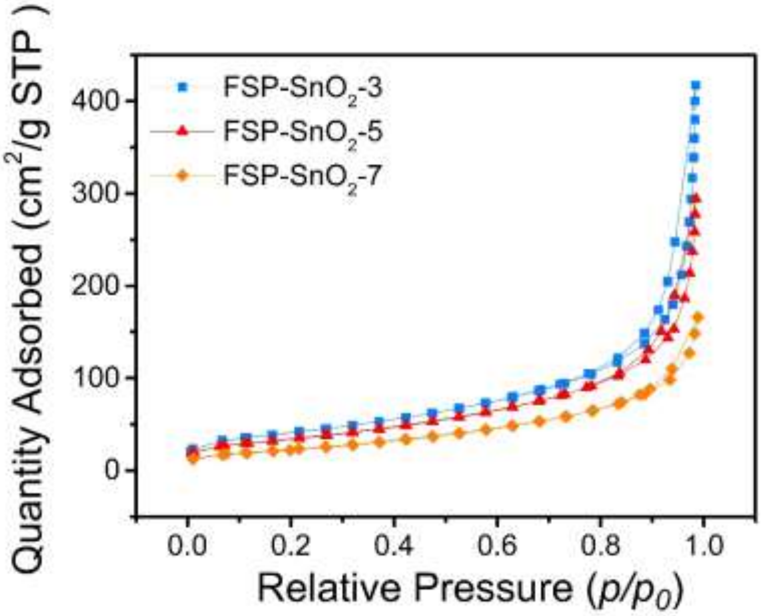

Figure S2. N_2 adsorption-desorption isotherms for FSP-SnO_2-3, FSP-SnO_2-5 and FSP-SnO_2-7. All the catalysts demonstrate a Type III isotherm that are consistent with reported FSP materials in literature. As the feed rate is increased, the catalysts demonstrate a decrease in surface area.

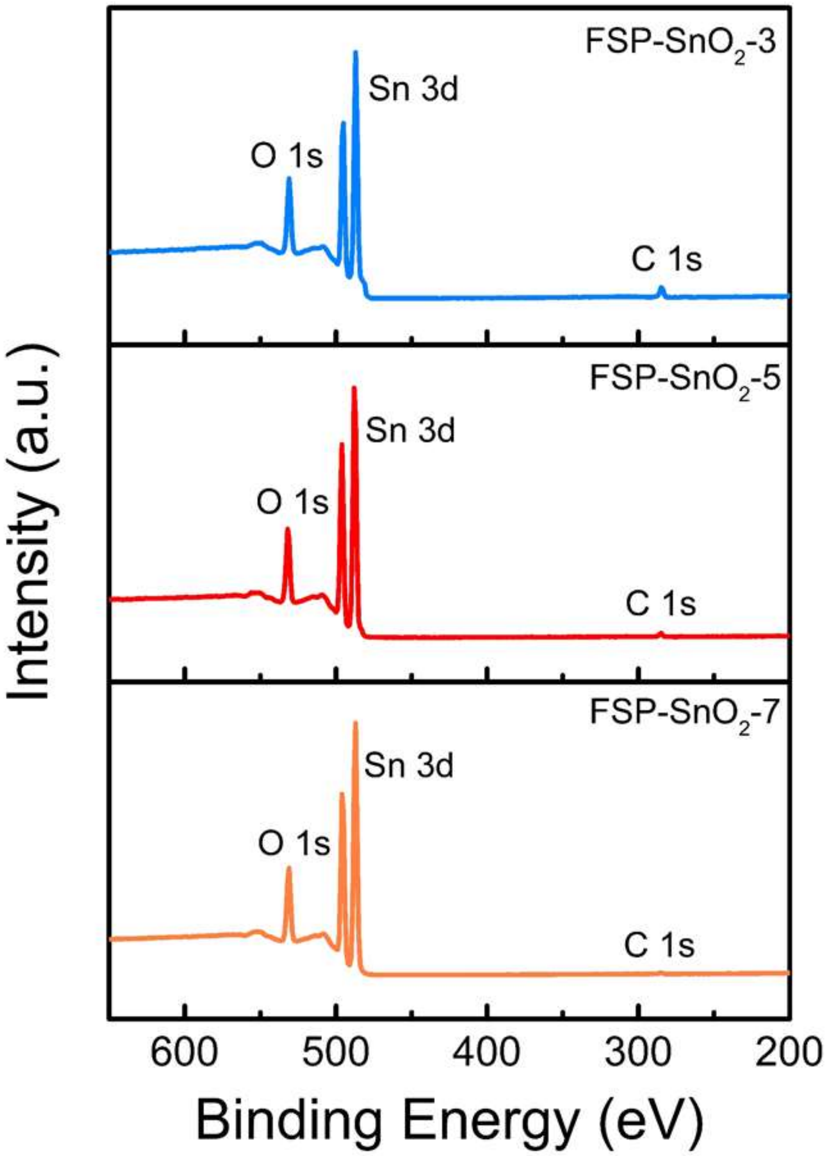

Figure S3. XPS survey spectrum for FSP SnO_2 catalysts. The survey spectrum confirmed the presence of Sn and O species within the as-synthesized catalysts.

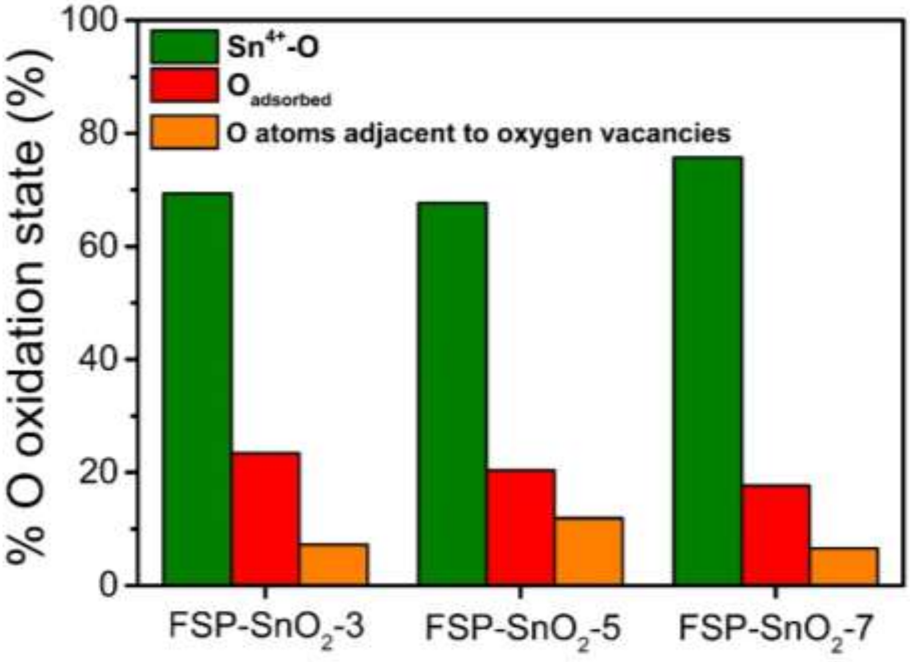

Figure S4. Variation of Sn^{4+}-O, $O_{adsorbed}$ and oxygen vacancy for FSP SnO_2 prepared at a feed rate of 3,5 and 7 mL/min. Increasing feed rate during FSP leads to a decrease in $O_{adsorbed}$ whereas the oxygen vacancy (represented by intensity of signals from O atoms adjacent to oxygen vacancies) was maximized for FSP-SnO_2-5.

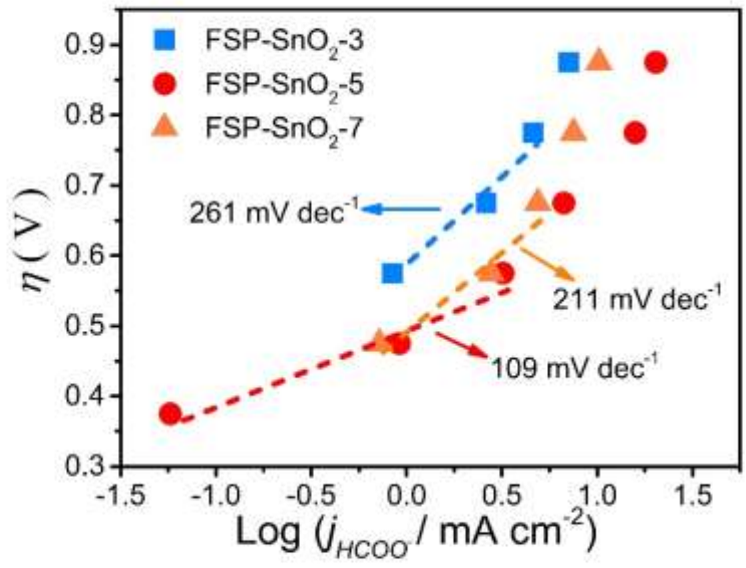

Figure S5. Tafel plots for $HCOO^-$ production on FSP-SnO_2-3, FSP-SnO_2-5 and FSP-SnO_2-7 in CO_2-saturated 0.1 M $KHCO_3$ solution. The mechanistic Tafel slopes for FSP-SnO_2-5 is 109 mV dec^{-1}, indicating faster reaction kinetics for the catalyst compared to FSP-SnO_2-7 (211 mV dec^{-1}) and FSP-SnO_2-3 (261 mV dec^{-1}), respectively.

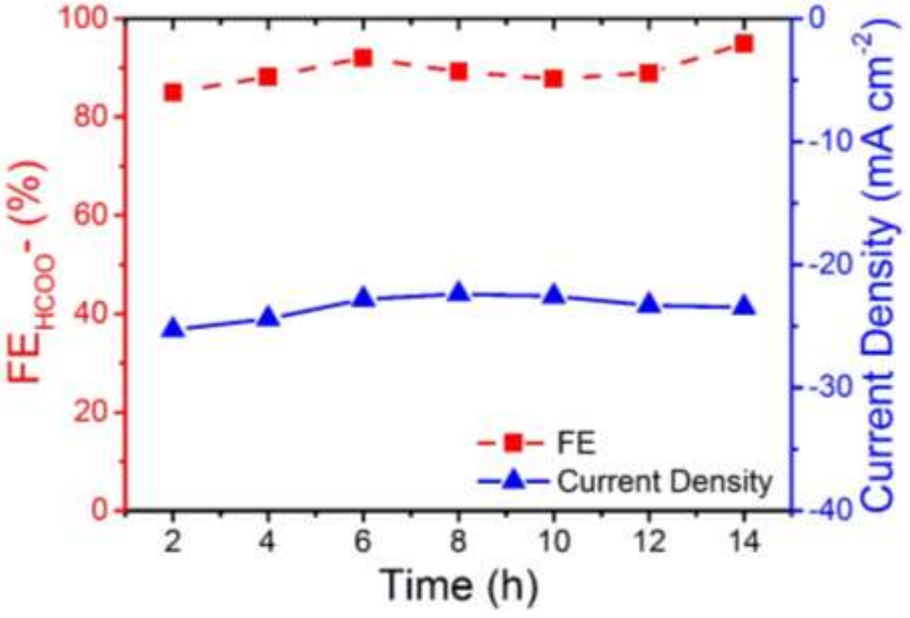

Figure S6. Long-term stability of FSP-SnO_2-5 at an applied potential of -1.1 V vs RHE in CO_2 saturated 0.1 M $KHCO_3$. The catalyst demonstrated prominent stability over the whole duration, indicating its practicality for large-scale application.

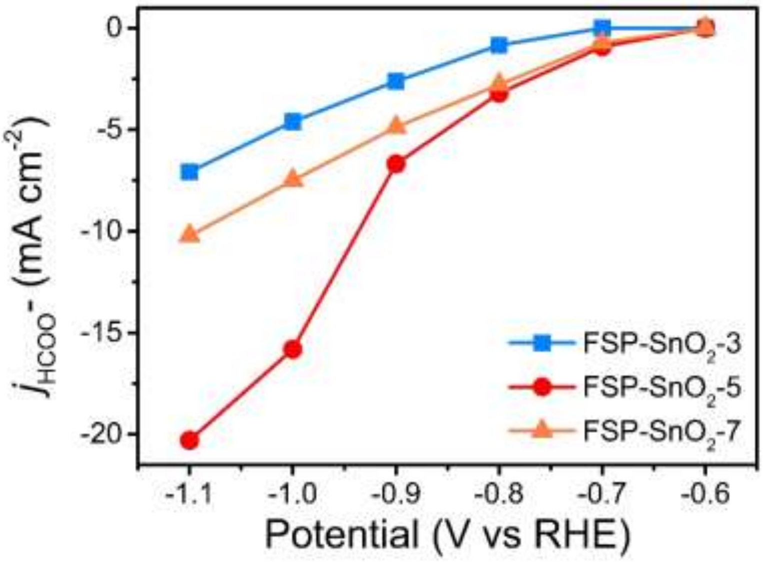

Figure S7. Partial current density for $HCOO^-$ generation for FSP-SnO_2 catalysts drop-casted on carbon paper under different applied potentials in CO_2 saturated 0.1 M $KHCO_3$. The partial current density was maximized for FSP-SnO_2-5. For instance, at the applied potential of -1.1 V, the measured j_{HCOO^-} for FSP-SnO_2-5 was -21 mA cm^{-2} compared to -7.3 mA cm^{-2} and -11 mA cm^{-2} attained with FSP-SnO_2-3 and FSP-SnO_2-7, respectively,

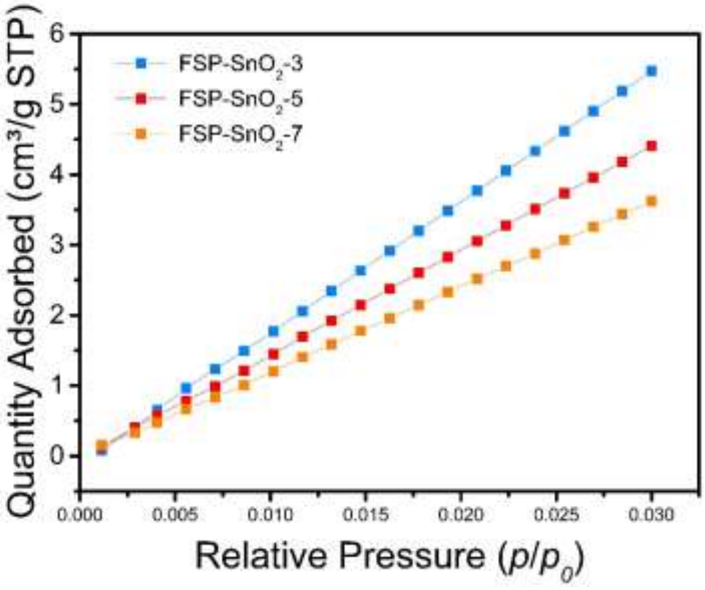

Figure S8. CO_2 BET results for FSP-SnO_2-3, FSP-SnO_2-5 and FSP-SnO_2-7. The collective results indicate that the physical adsorption of CO_2 decreases with increasing feed-rate during FSP, indicating that the physical adsorption of CO_2 is reliant on the surface area and therefore does not show any direct correlation with FE_{HCOO^-} or j.

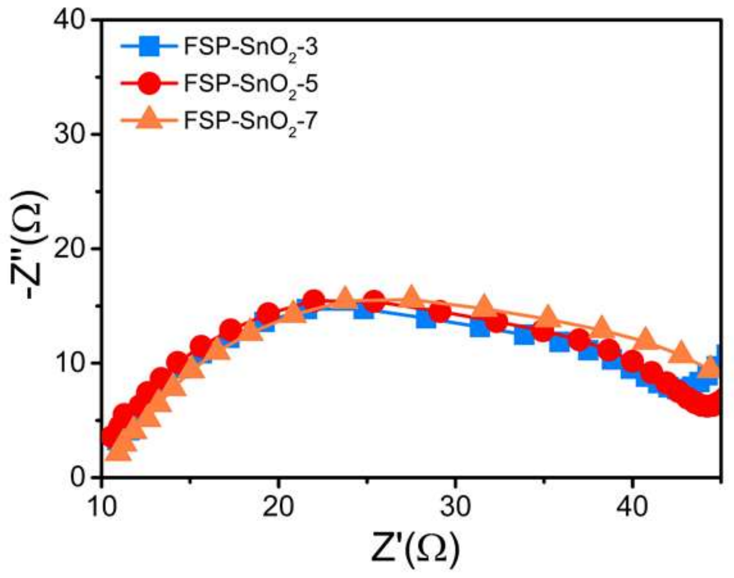

Figure S9. **EIS measurements at -1.1 V vs RHE for FSP-SnO_2 catalysts in CO_2 saturated 0.1 M $KHCO_3$ solution.** The Nyquist plots indicate that the radius of the semicircle for FSP-SnO_2 catalysts are alike, indicating similar impedance (electrode conductivity and charge transfer resistance).

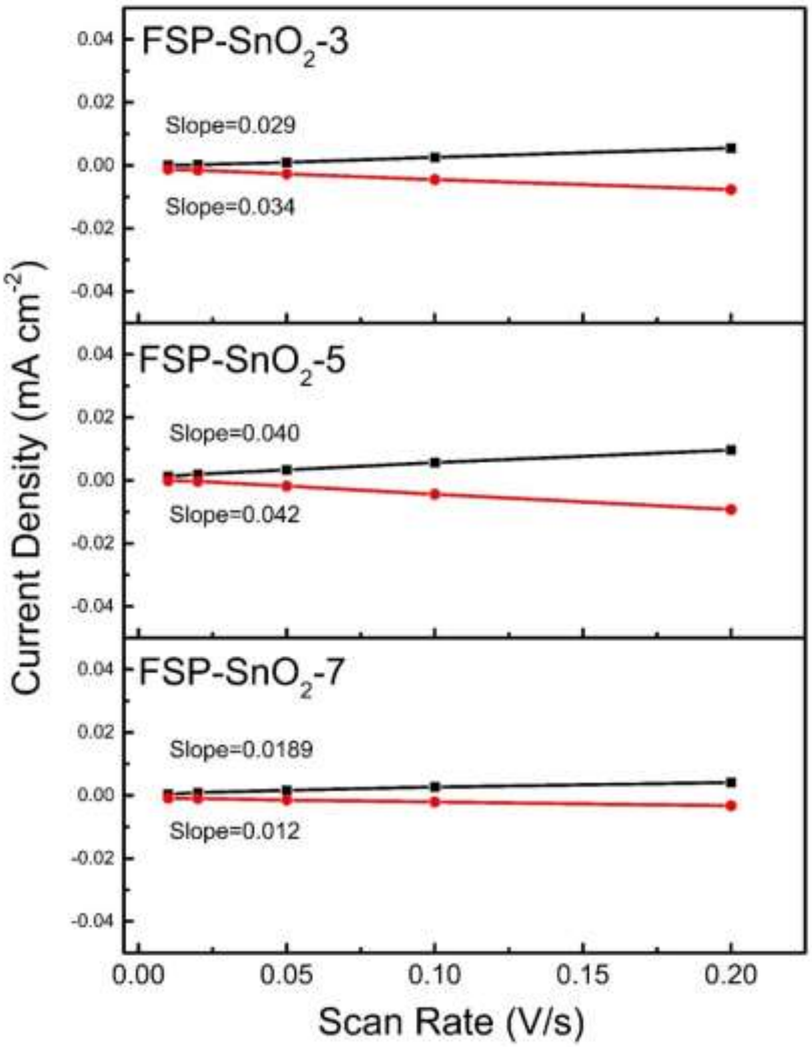

Figure S10. ECSA measurements. Current density plots at various Cyclic Voltammetry (CV) scan rates for FSP-SnO_2 catalysts loaded on carbon black support. Non-Faradaic charging currents were first measured in the potential range of +0.4 V and +0.2 V vs RHE as the scan rate was varied between 10, 20, 50, 100 and 200 mV/s. The cathodic (negative) and anodic (positive) current densities were obtained from the double layer charge/discharge curves at +0.30 V vs RHE. The double layer capacitance was then calculated by averaging the absolute values of cathodic and anodic slopes of the linear fits. The slope obtained with FSP-SnO_2-3, FSP-SnO_2-5 and FSP-SnO_2-7 are 0.03 mF/cm^2, 0.04 mF/cm^2 and 0.02 mF/cm^2, respectively indicating that the variation in ECSA is insignificant.

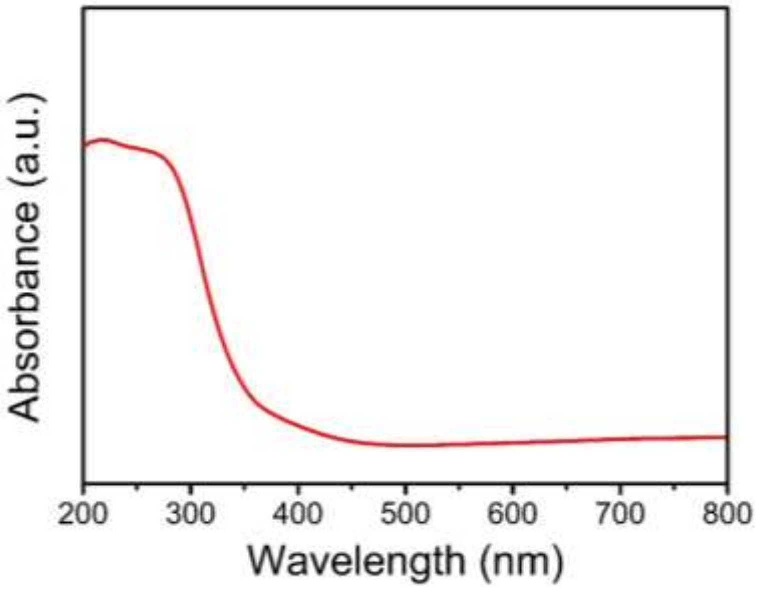

Figure S11. UV-vis diffuse reflectance spectra with FSP-SnO_2-5. The spectra does not display any features associated with defects, suggesting that the electronic states of FSP-SnO_2-5 is not affected as a result of defects.

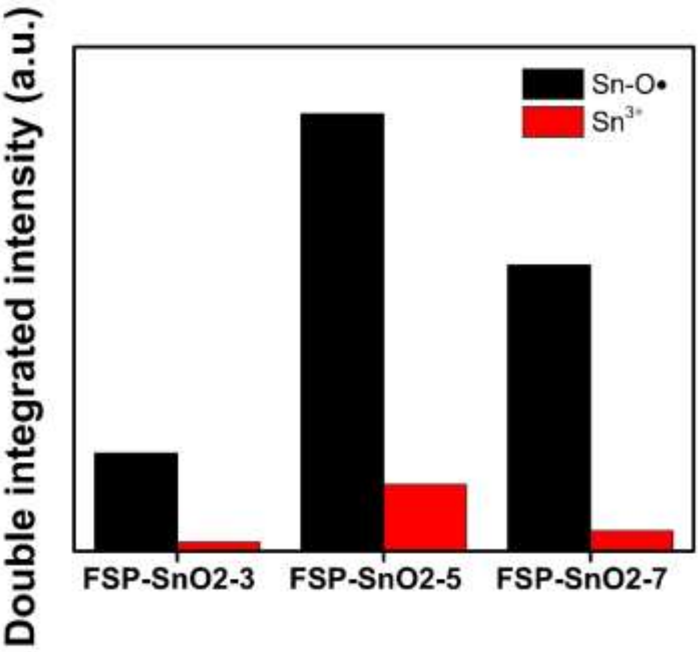

Figure S12. Double integrated intensity of EPR spectra for FSP SnO_2 prepared at a feed rate of 3, 5, and 7 ml/min. The experimental EPR spectrum is the first derivative of absorption with respect to the magnetic field. Double integration method is one of the best technique to estimate the area under the EPR signal which is proportional to the number of spins and amount of defects when the signal of interest can be separated from the background as well as overlapping signals. [1] It can be observed from Figure S11 that the intensity of oxygen hole centers (OHC) and oxygen vacancies arising from Sn^{3+} is maximized for FSP-SnO_2-5 followed by FSP-SnO_2-7 and FSP-SnO_2-3, corresponding well with the observed electrocatalytic activity of the catalysts for CO_2RR.

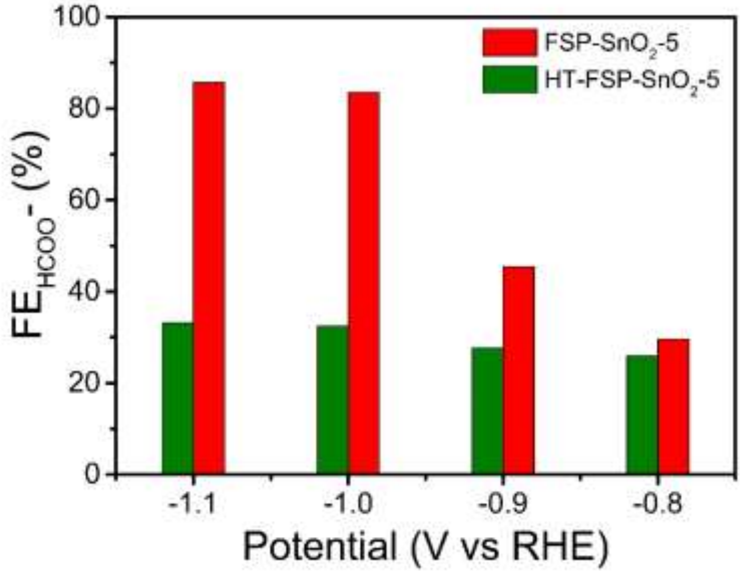

Figure S13. Formate selectivity for heat-treated FSP-SnO_2-5 compared with FSP-SnO_2-5. Removal of defects by annealing FSP-SnO_2-5 in air leads to a decrease in FE_{HCOO^-} compared to FSP-SnO_2-5, highlighting the necessity of defects in governing CO_2RR to formate.

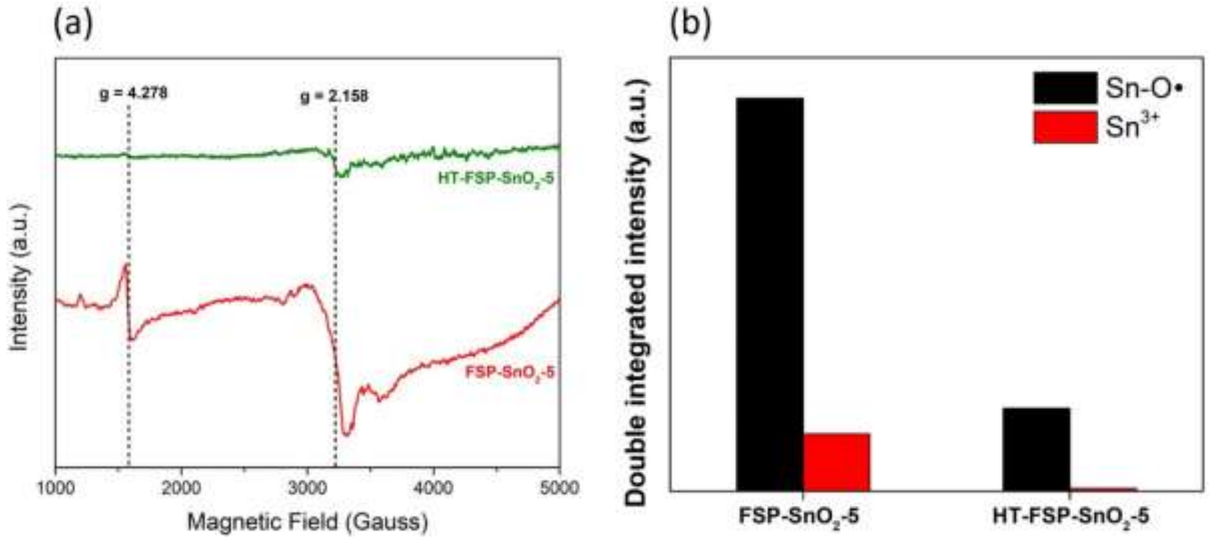

Figure S14. Defect removal as a result of heat-treatment with FSP-SnO_2-5 in air for duration of 5 hours. (a) EPR spectra and **(b)** their corresponding double integrated intensity of FSP SnO_2-5 and HT-FSP SnO_2-5.

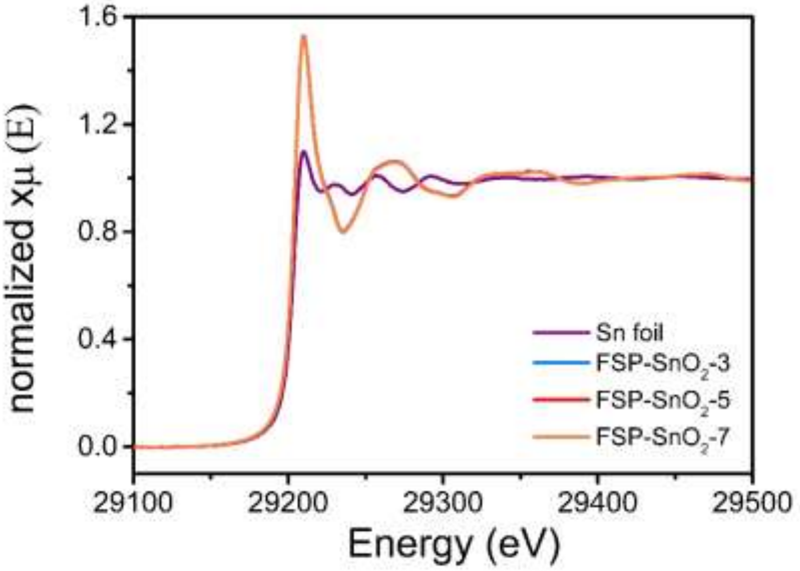

Figure S15. XANES for FSP SnO_2 prepared at a various feed rate. The Sn K-edge XANES spectra for FSP-SnO_2-3 (blue), FSP-SnO_2-5 (red) and FSP-SnO_2-7 (orange) overlap, indicating similar bulk electronic structure of the as-synthesized catalysts. For reference, a Sn foil (purple) is also presented as well.

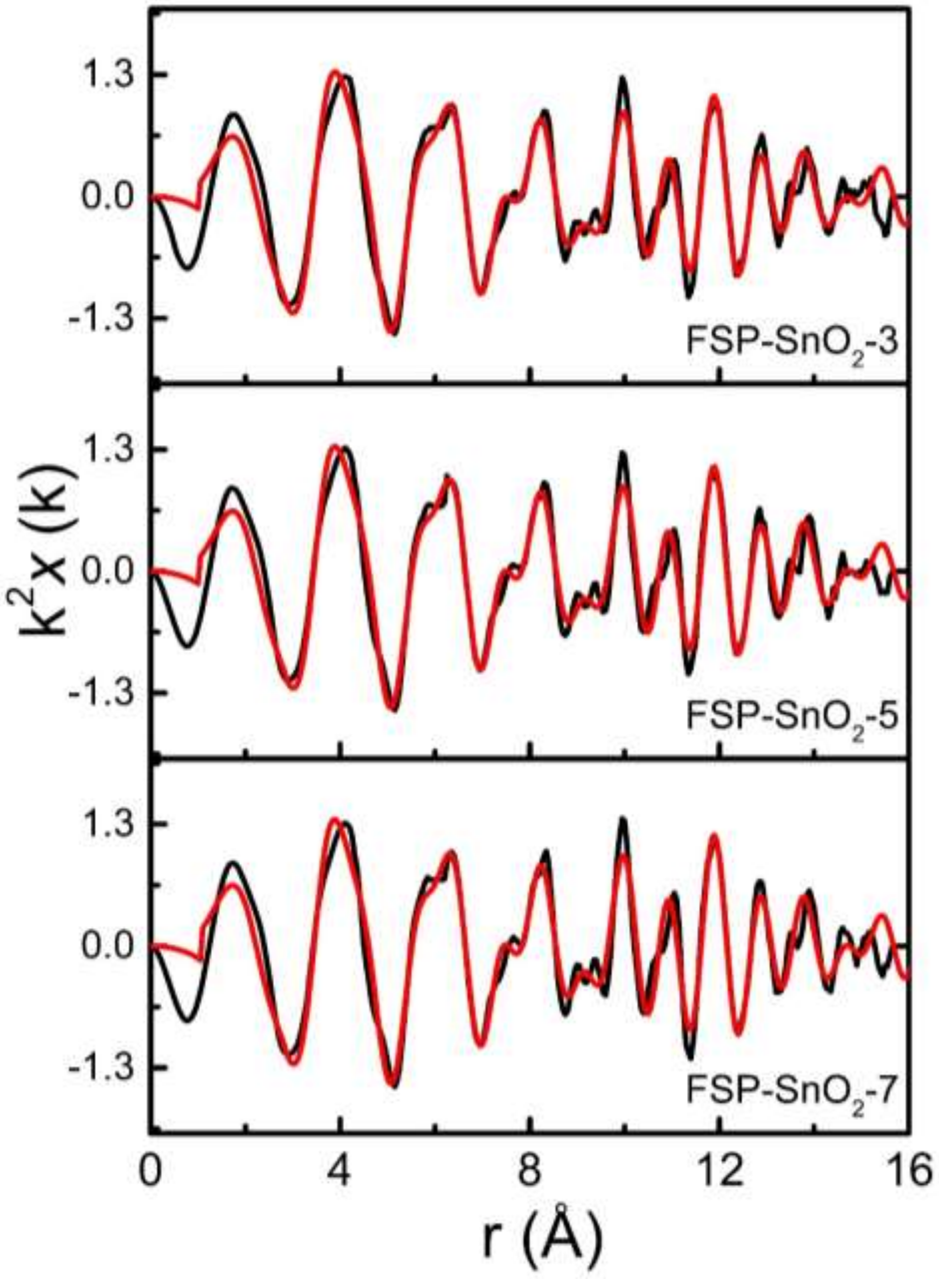

Figure S16. EXAFS oscillation function k^2 *x* (k) for FSP-SnO_2-3, FSP-SnO_2-5 and FSP-SnO_2-7. Note that the red line indicates fitted data.

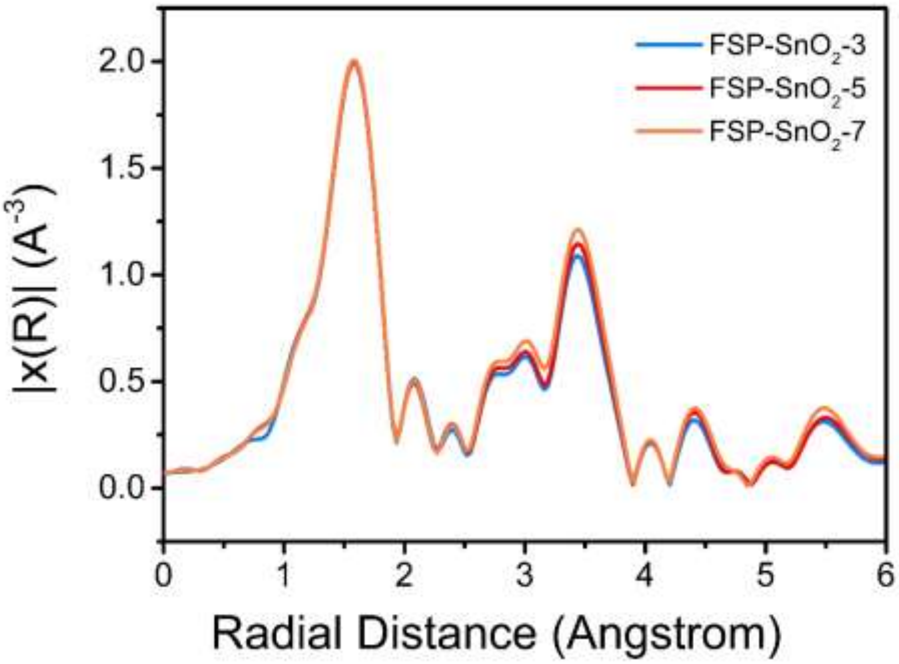

Figure S17. Sn K-edge EXAFS for FSP SnO_2 prepared at a feed rate of 3,5 and 7 mL/min. The EXAFS spectra of the FSP catalysts are similar, suggesting that a similar atomic environment is shared amongst the Sn species. These results indicate that the defects present in FSP-SnO_2 catalysts are on the surface and as such, the low concentration of defects is not picked up by Sn K-edge EXAFS measurements.

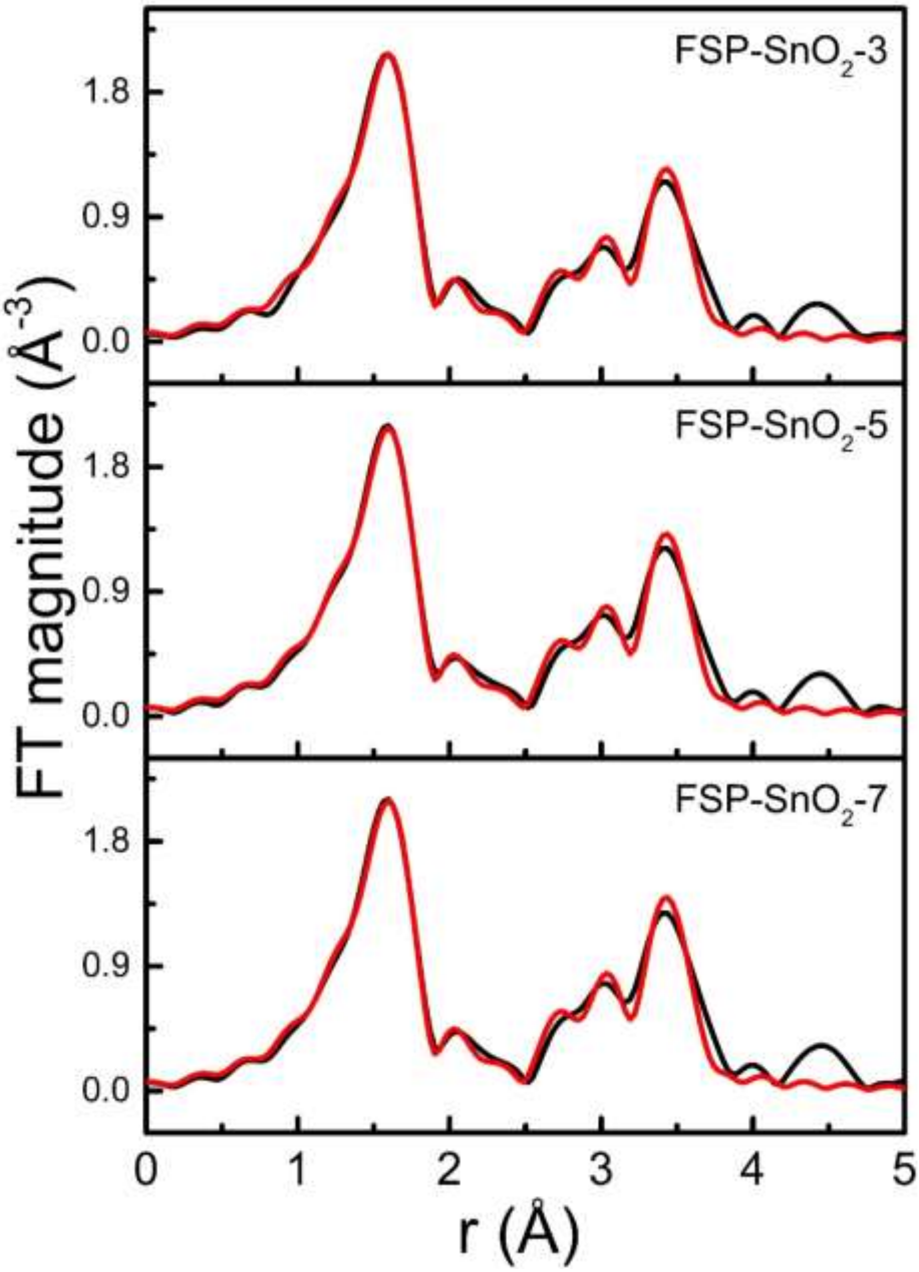

Figure S18. Fitted EXAFS data in R space for FSP-SnO_2-3, FSP-SnO_2-5 and FSP-SnO_2-7. Note that the red line indicates fitted data.

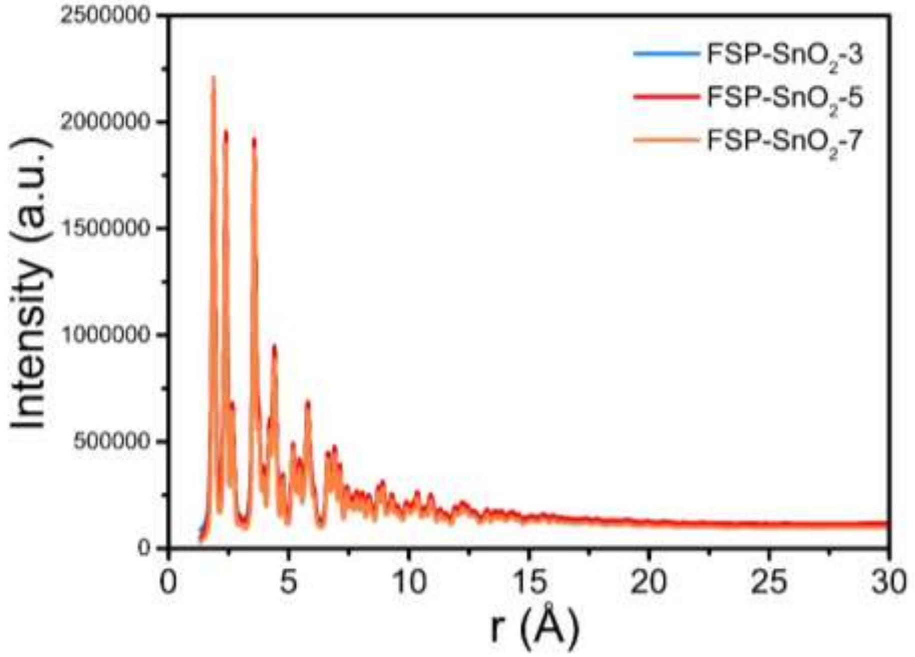

Figure S19. HE-XRD plots for FSP-SnO_2 catalysts prepared at a feed rate of 3,5 and 7 mL/min. The high-resolution XRD plots are transformed into structure factors (Figure S19) and subsequently Fourier transformed into atomic pair distribution function.

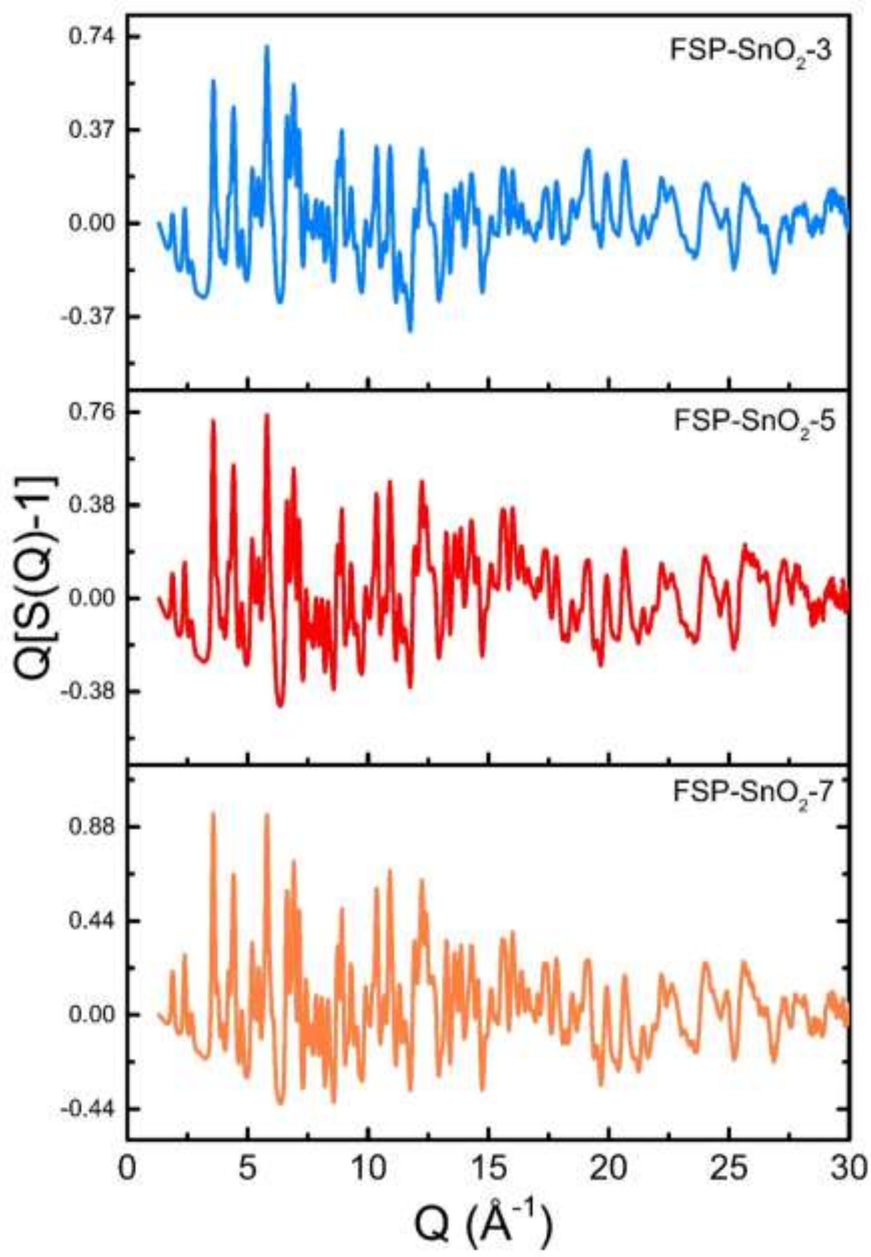

Figure S20. Reduced structure factors of FSP-SnO_2 catalysts prepared at a feed rate of 3,5 and 7 mL/min. The reduced structure factors were Fourier transformed into atomic pair distribution functions using the program RAD.

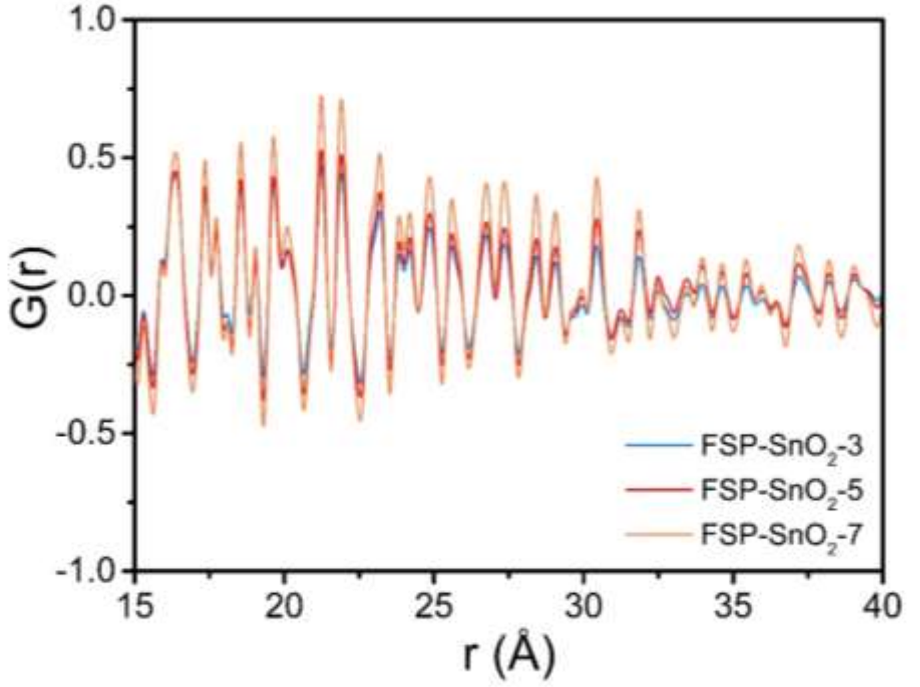

Figure S21. Atomic pair distribution function for FSP-SnO_2-3, FSP-SnO_2-5 and FSP-SnO_2-7 past 15 Å. The results showcase the diminished atomic pair oscillations as a function of radial distance.

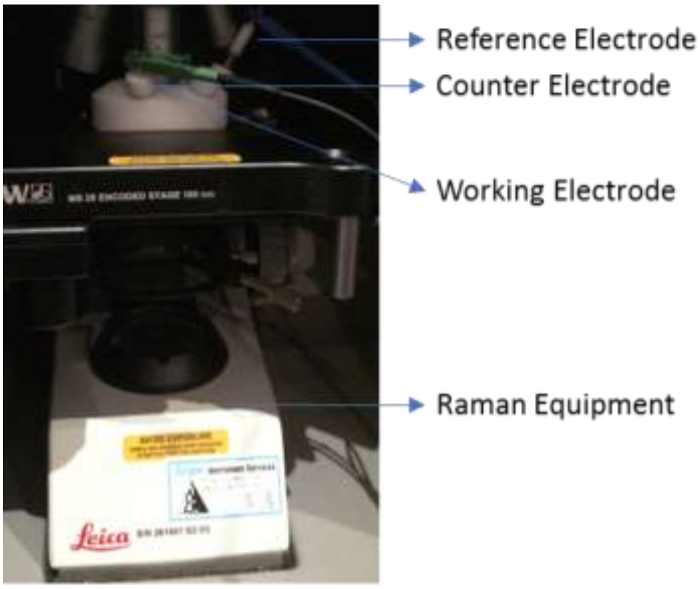

Figure S22. In-situ Raman experiment setup for CO_2RR. FSP-SnO_2-5 ink was drop-casted on glassy carbon electrode and was placed in CO_2 saturated 0.1 M $KHCO_3$ solution where SCE reference and Pt counter electrode were utilized. Raman spectra was first obtained when no potential was applied to establish the background. Subsequently, Raman measurements were carried out when the applied potential was -1.5 V (for 300 seconds) vs RHE to study the change in surface chemical states for the catalyst during CO_2RR.

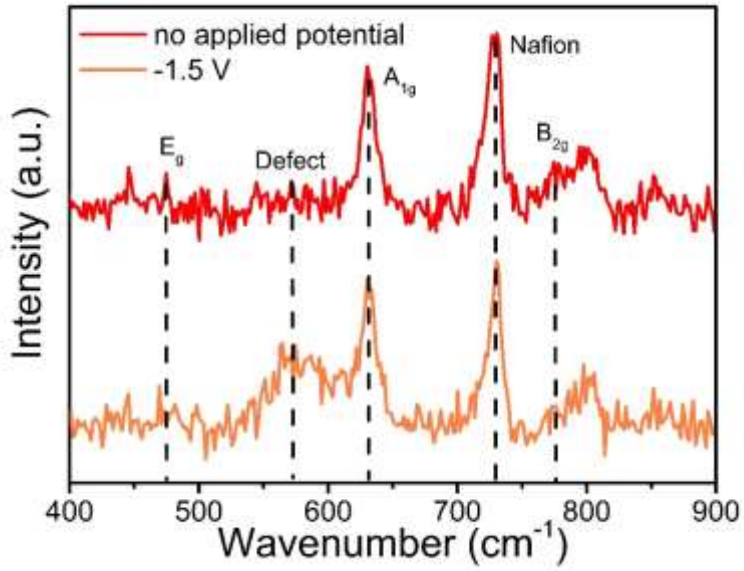

Figure S23. In-situ Raman spectra for FSP-SnO_2-5 under experimental CO_2RR conditions. The Raman spectra clearly indicated that the application of potential does not lead to complete reduction of SnO_2 species (as is evident by strong Raman $A_{1g,}$ B_{2g} and E_g peaks for SnO_2). Moreover, the application of potential leads to an increase in peak signal corresponding to surface defects, indicating the presence of such species on SnO_2 under applied bias.

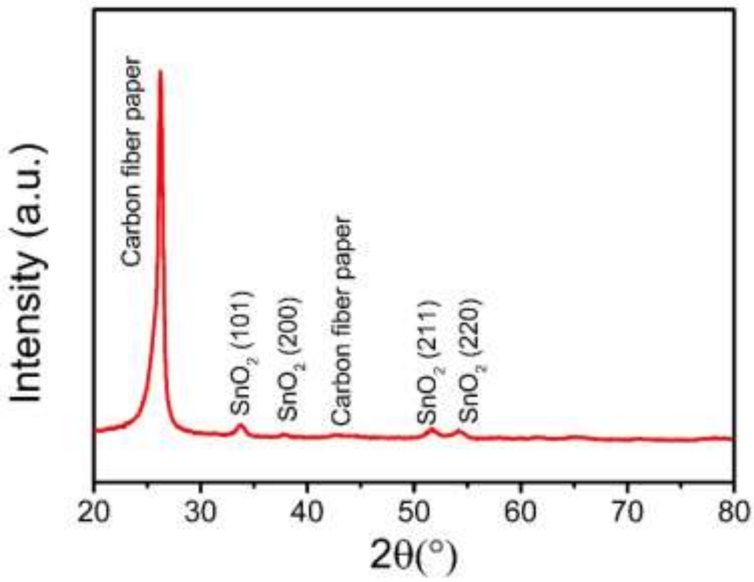

Figure S24. Post-reaction XRD pattern of FSP-SnO_2-5 electrode. The XRD pattern demonstrates the presence of SnO_2 species after long-term CO_2RR experiments indicating high structural stability.

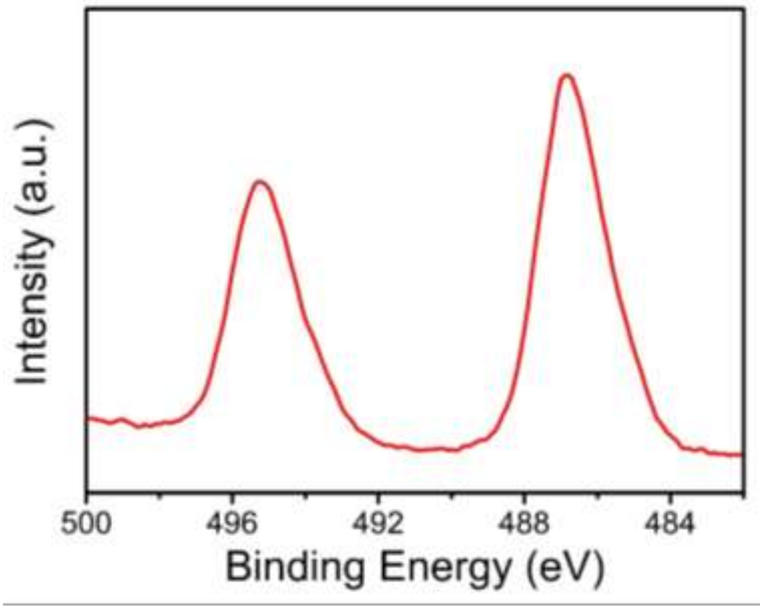

Figure S25. Post-reaction Sn 3d XPS spectra for FSP-SnO_2-5 electrode. The ex-situ XPS spectra indicates the presence of SnO_2 after CO_2RR.

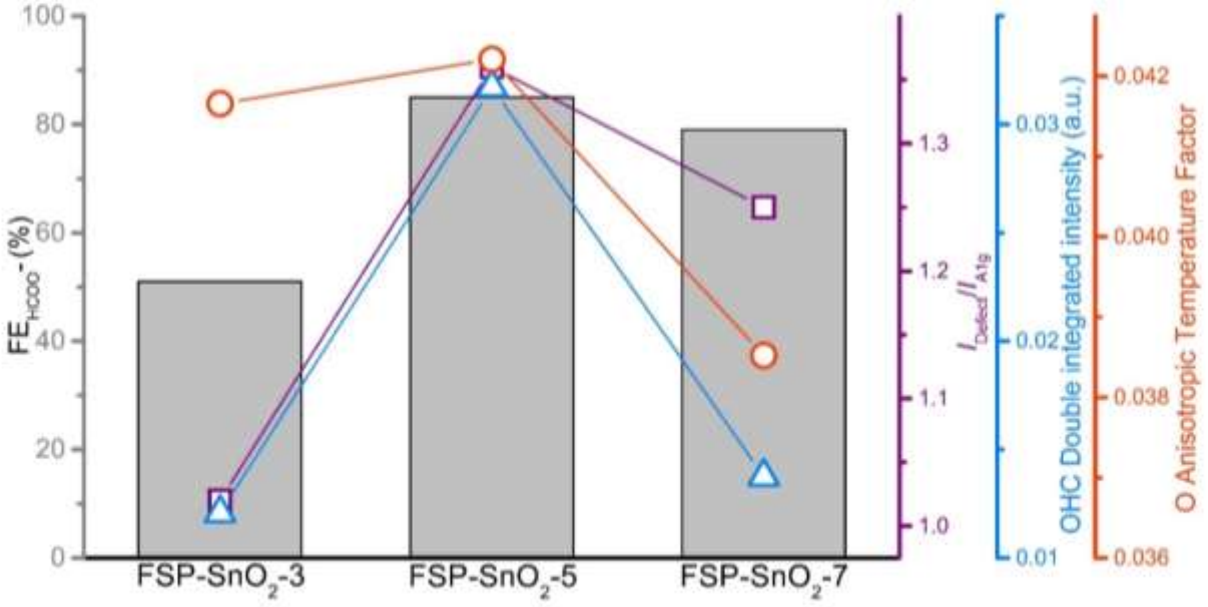

Figure S26. Defect-activity relationship for FSP-catalysts. Defect densities were calculated using I_{defect}/I_{A1g}, OHC double integrated intensity and O anisotropic temperature factor. The electrocatalytic results were obtained at an applied potential of -1.1 V.

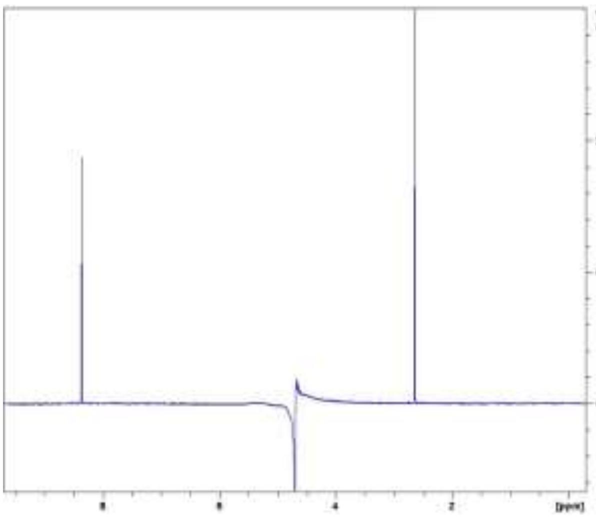

Figure S27. Typical 1D ^{1}H spectrum obtained with NMR analysis on liquid aliquots taken after CO_2RR.

Supplementary Tables

Table S1. Summary of surface area and particle diameter sizes for FSP-SnO_2 catalysts.

Sample	SSA_{BET} (m^2/g)	d_{BET} (nm)	d_{XRD} (nm)	d_{TEM} (nm)
**FSP-SnO_2-3**	146	5.9	5.7	8.6
**FSP-SnO_2-5**	121	7.1	7.3	10.1 - 12
**FSP-SnO_2-7**	81.4	10.6	11.8	14.2

Table S2. CO_2RR catalytic performances of various Sn-based catalysts in similar operating conditions for $HCOO^-$ production.

Catalyst	Electrolyte	Operating Potential (V vs RHE)	Current Density (mA/cm^2)	Faradaic Efficiency for $HCOO^-$ (%)	Reference
**FSP-SnO_2-7**	**0.1 M $KHCO_3$**	**-1.1**	**23.7**	**85**	**This Work**
m-SnO_2	0.1 M $KHCO_3$	-1.15	10.8	75.2	[2]
An-Sn_{30}	0.1 M $KHCO_3$	-1.09	4.80	77.4	[3]
SnO_2/Graphene	0.1M $NaHCO_3$	-1.16	10.2	93.6	[4]
1D SnO_2 Wire in Tube	0.1 M $KHCO_3$	-0.99	7	63	[5]
SnO_2/C hollow sphere	0.1 M $KHCO_3$	-0.9	11	54.2	[6]
Heat-treated Sn dendrite	0.1 M $KHCO_3$	–1.36	17.1	71.6	[7]
Sn Foam	0.1 M $NaHCO_3$	-1.3	23.5	90	[8]
Sn Foil	0.5 M Na_2SO_4	-1.4	5	75.92	[9]
Sn Plate	0.1M $KHCO_3$	-1.2	7	75	[10]
Sn Foil	0.5 M KOH	-0.95	0.5	69.5	[11]
$Sn_6O_4(OH)_4$ NPs SnO_2 NPs	0.1M K_2SO_4	-1.2	N/A	75	[12]
Sn/SnOx/Ti	0.5 M $NaHCO_3$	-0.7	1.8	55	[13]
Annealed Sn NPs	0.1M $KHCO_3$	-1.2	4	51.5	[14]
Sn NPs (GDE)	0.5 M $NaHCO_3$	-1.2	27	70	[15]
SnO_2 Nanospheres (GDE)	0.5 M $KHCO_3$	-1.1	6	68	[16]
Sn quantum sheets/Graphene	0.1 M $NaHCO_3$	-1.15	21.5	89	[17]
SnO_2/MWCNT	0.5 M $NaHCO_3$	-1.05	~80	26	[18]
SnO_2 microsphere (GDE)	0.5 M $KHCO_3$	-1.3	12.5	62	[19]
SnO_2 Nanowires (Plasma Treated)	0.1 M $KHCO_3$	-0.8	6	81	[20]
SnO_2 nanosheets on carbon cloth	0.5 M $NaHCO_3$	-0.99	45	87	[21]

Table S3. EXAFS curve-fitting results.

Sample	Sn-O_1 CN	Sn-O_1 NND (Å)	Sn-O_1 σ^2 (Å^2)	Sn-Sn_1 CN	Sn-Sn_1 NND (Å)	Sn-Sn_1 σ^2 (Å^2)	Sn-O_2 CN	Sn-O_2 NND (Å)	Sn-O_2 σ^2 (Å^2)	Sn-Sn_2 CN	Sn-Sn_2 NND (Å)	Sn-Sn_2 σ^2 (Å^2)
FSP-SnO_2-3	5.11 ± 0.24	2.055 ± 0.006	0.003 ± 0.001	2.39 ± 0.39	3.196 ± 0.008	0.003 ± 0.001	3.83 ± 0.41	3.53 ± 0.04	0.003 ± 0.001	5.41 ± 0.57	3.726 ± 0.006	0.003 ± 0.001
FSP-SnO_2-5	5.12 ± 0.25	2.055 ± 0.006	0.003 ± 0.001	2.55 ± 0.40	3.200 ± 0.008	0.003 ± 0.001	4.01 ± 0.42	3.53 ± 0.04	0.003 ± 0.001	5.70 ± 0.59	3.726 ± 0.006	0.003 ± 0.001
FSP-SnO_2-7	5.14 ± 0.28	2.056 ± 0.007	0.003 ± 0.001	2.63 ± 0.42	3.197 ± 0.008	0.003 ± 0.001	4.59 ± 0.48	3.54 ± 0.04	0.003 ± 0.001	5.93 ± 0.62	3.727 ± 0.006	0.003 ± 0.001

Table S4. Fitted lattice parameters from PDF measurement for FSP-SnO_2 catalysts.

Catalyst	Lattice Parameters		
	a (Å)	b (Å)	c (Å)
FSP-SnO_2-3	4.72131	4.72131	3.1774
FSP-SnO_2-5	4.72295	4.72295	3.17802
FSP-SnO_2-7	4.7234	4.7234	3.17724

Table S5. Anisotropic temperature factors for FSP-SnO_2 catalysts.

Catalyst	Sn	O
FSP-SnO_2-3	0.0079899	0.0416578
FSP-SnO_2-5	0.00798129	0.0422125
FSP-SnO_2-7	0.00759314	0.0385221

References

[1] C. G. R. Eaton, D. G. Gorenstein, *Bull. Magn. Reson.* **1988**, *10*, 130.
[2] R. Daiyan, X. Lu, W. H. Saputera, Y. H. Ng, R. Amal, *ACS Sustain. Chem. Eng.* **2018**, *6*, 1670.
[3] R. Daiyan, X. Lu, Y. H. Ng, R. Amal, *Catal. Sci. Technol.* **2017**, *7*, 2542.
[4] S. Zhang, P. Kang, T. J. Meyer, *J. Am. Chem. Soc.* **2014**, *136*, 1734.
[5] L. Fan, Z. Xia, M. Xu, Y. Lu, Z. Li, *Adv. Funct. Mater.* **2018**, *28*, 1.
[6] Y. Yiliguma, Z. Wang, C. Yang, A. Guan, L. Shang, A. M. Al-Enizi, L. Zhang, G. Zheng, *J. Mater. Chem. A* **2018**.
[7] D. H. Won, C. H. Choi, J. Chung, M. W. Chung, E.-H. Kim, S. I. Woo, *ChemSusChem* **2015**, *8*, 3092.
[8] D. Du, R. Lan, J. Humphreys, S. Sengodan, K. Xie, H. Wang, S. Tao, *ChemistrySelect* **2016**, *1*, 1711.
[9] J. Wu, F. G. Risalvato, F.-S. Ke, P. J. Pellechia, X.-D. Zhou, *J. Electrochem. Soc.* **2012**, *159*, F353.
[10] W. Lv, R. Zhang, P. Gao, L. Lei, *J. Power Sources* **2014**, *253*, 276.
[11] H.-Y. Kim, I. Choi, S. H. Ahn, S. J. Hwang, S. J. Yoo, J. Han, J. Kim, H. Park, J. H. Jang, S.-K. Kim, *Int. J. Hydrogen Energy* **2014**, *39*, 16506.
[12] M. F. Baruch, J. E. Pander, J. L. White, A. B. Bocarsly, *ACS Catal.* **2015**, *5*, 3148.
[13] Y. Chen, M. W. Kanan, *J. Am. Chem. Soc.* **2012**, *134*, 1986.
[14] J. Wu, F. G. Risalvato, S. Ma, X.-D. Zhou, *J. Mater. Chem. A* **2014**, *2*, 1647.
[15] G. K. S. Prakash, F. A. Viva, G. A. Olah, *J. Power Sources* **2013**, *223*, 68.
[16] D. Universtiy, N. Road, S. Carolina, U. States, **2015**, *66*, 53.
[17] F. Lei, W. Liu, Y. Sun, J. Xu, K. Liu, L. Liang, T. Yao, B. Pan, S. Wei, Y. Xie, *Nat. Commun.* **2016**, *7*, 12697.
[18] S. Bashir, S. S. Hossain, S. U. Rahman, S. Ahmed, A. Al-Ahmed, M. M. Hossain, *J. CO2 Util.* **2016**, *16*, 346.
[19] Y. Fu, Y. Li, X. Zhang, Y. Liu, J. Qiao, J. Zhang, D. P. Wilkinson, *Appl. Energy* **2016**, *175*, 536.
[20] B. Kumar, V. Atla, J. P. Brian, S. Kumari, T. Q. Nguyen, M. Sunkara, J. M. Spurgeon, *Angew. Chemie - Int. Ed.* **2017**, *56*, 3645.
[21] F. Li, L. Chen, G. P. Knowles, D. R. MacFarlane, J. Zhang, *Angew. Chemie Int. Ed.* **2017**, *56*, 505.
